# Microglial Adaptations to Chronic Nicotine in the Cerebellum: Proteomic Evidence for Neuroimmune Vulnerability

**DOI:** 10.1021/acs.jproteome.5c01027

**Published:** 2026-03-30

**Authors:** Aya Nusir, Scott M. Anthony, Weidong Zhou, Nadine Kabbani

**Affiliations:** † Interdisciplinary Program in Neuroscience, School of Systems Biology, 3298 George Mason University, Fairfax, Virginia 22030, United States; ‡ Biomedical Research Laboratory, 3298 George Mason University, 10650 Pyramid Place, Manassas, Virginia 20110, United States; § Center for Applied Proteomics and Molecular Medicine, George Mason University, 10920 George Mason Circle, Manassas, Virginia 20110, United States

**Keywords:** addiction, smoking, inflammation, neurodegeneration, sex differences

## Abstract

Smoking is a major public health concern with widespread effects on multiple organ systems, including the immune system. Chronic nicotine exposure can alter immune cell function through nicotinic receptors expressed on peripheral macrophages and microglia in the brain. Recent evidence indicates that the cerebellum is impacted by nicotine, contributing to motor and nonmotor outcomes, during drug use. In this study, we investigated the effect of chronic nicotine on microglia proteomes in the adult mouse cerebellum. Microglia were isolated by fluorescence-activated cell sorting (FACS) based on CD11b^high^ CD45^low/intermediate^ expression from male and female mice (*n* = 9 per group) exposed to 200 μg/mL nicotine (dissolved in 2% saccharin drinking water) for 30 days. Proteomic analysis was performed using liquid chromatography electrospray ionization (LC-ESI) mass spectrometry (MS) comparing the effect of nicotine relative to vehicle control. Our results reveal a sex-dependent effect of nicotine on microglial proteomes. While both males and females exhibited histone-related genomic responsiveness to nicotine, males demonstrated enrichment in cytoskeletal and metabolic proteins, and females showed complement-protein adaptations. The microglial proteome in male mice displayed nicotine-related adaptations in proteins that can contribute to neurodisorders including Huntington’s disease and amyotrophic lateral sclerosis (ALS), of which smoking is a known risk factor. Together, these results highlight an effect of nicotine on the proteome of microglia providing insight into immune pathways that can contribute to smoking-related behavior and disease.

## Graphical abstract

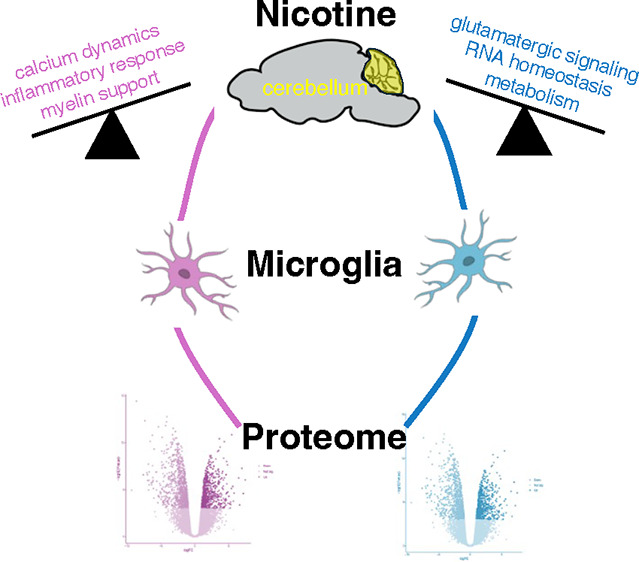

## Introduction

Nicotine is a highly addictive substance exerting cognitive and emotional regulatory effects through interaction with nicotinic acetylcholine receptors (nAChRs) that are expressed throughout the mammalian brain on neurons and glia.^1,2^ These receptors influence neural development, synaptic plasticity, and neuroimmune signaling.^3–6^ While the systemic effects of nicotine are well documented, emerging evidence indicates that chronic nicotine use alters neuroimmune function through its actions on microglia and macrophages.^7,8^ Microglia are the resident immune cells of the brain and play an important role in synaptic development and maintenance.^9^ Microglia are heterogeneous cells whose functional states vary across brain regions and are influenced by hormonal regulation; these differences are increasingly recognized as determinants of disease vulnerability and potential modifiers of therapeutic outcomes.^10,11^

Nicotine modulates several important signaling pathways through the activation of nAChRs, particularly the α7 type that are expressed within microglia.^2^ The activation of nAChRs leads to calcium influx and downstream regulation of cytokine production (e.g., TNF-α, IL-1β), shifting microglia between anti- (M2) or pro-inflammatory (M1) phenotypes depending on dose and chronicity.^12–14^ In addition, nicotine has been shown to reduce cAMP levels and increase p38 phosphorylation while driving transcriptional reprogramming through the JAK/STAT pathway.^15–17^ Together, these processes indicate that nicotine exposure can alter the activation of microglia, influencing their interactions with neurons and astrocytes, modulating synaptic plasticity, and contributing to outcomes associated with addiction and neurodegeneration.^8,18^

The cerebellum is increasingly recognized as a participant in reward-related circuitry and a contributor to nicotine dependence and withdrawal.^19–21^ The cerebellum is organized into a highly conserved cytoarchitecture, consisting of Purkinje cells, granule cells, and interneurons layered in a manner that supports precise motor coordination as well as cognitive and affective processing. Within the cerebellum, microglia exhibit functional properties that contrast those of microglia in other brain regions such as the cortex.^22^ For example, cerebellar microglia are relatively sparse, exhibit greater somatic motility, and demonstrate strong sexual dimorphism in their morphology and signaling responses.^23^ Cerebellar microglia alsod high turnover rates and phagocytotic activity when compared to cortical microglia.^23–25^

Here, we investigated how chronic nicotine exposure alters cerebellar microglial proteomes using fluorescence-activated cell sorting (FACS) and mass spectrometry–based proteomics in adult male and female mice following 30 days of nicotine exposure. The results reveal sex-specific adaptations in metabolic, cytoskeletal, and immune pathways, providing new insight into how nicotine modulates cerebellar neuroimmune function and vulnerability to smoking-related neurodisease.

## Methods

### Animal Husbandry, Drug Administration, and Tissue Collection

Male and female C57BL/6J mice (8 weeks old) were purchased from The Jackson Laboratory and housed under standard conditions on a 12-h light/dark cycle (lights off at 11:00 AM) with ad libitum access to food and water. Mice were group-housed, handled daily and allowed to acclimate for 1 week prior to treatment assignment. Mice were moved to single housing for the remainder of the treatment after random assignment to either the nicotine or vehicle group (*n* = 9 per sex per condition). Nicotine administration was performed as described.^26^ Briefly, nicotine (200 μg/mL) was prepared daily in 2% (w/v) saccharin water and provided as the sole source of drinking fluid for 30 consecutive days, and control animals received 2% (w/v) saccharin water alone. Fluid consumption and body weight were monitored twice weekly throughout the treatment period. At the conclusion of the 30-day treatment, animals were anesthetized with isoflurane and euthanized by decapitation. Brains were removed and cerebella were dissected on ice. Cerebellum tissue from each animal was weighed before flash freezing and sample pooling along each experimental group: nicotine-treated male, nicotine-treated female, saccharin-treated male, saccharin-treated female. All procedures were approved by the Yale University Institutional Animal Care and Use Committee (IACUC protocol #2025-07895) and were carried out in compliance with the National Institute of Health’s Guide for the Care and Use of Laboratory Animals.

### Cell Preparation

Tissue was mechanically dissociated in ice-cold Hanks’ Balanced Salt Solution (HBSS, calcium and magnesium-free; Gibco, Cat# 14175079) supplemented with protease and phosphatase inhibitor cocktail (Thermo Fisher, Cat# 78425) using a prerinsed glass Dounce homogenizer. The homogenate was passed through a 70 μm cell strainer and centrifuged at 400 × g for 5 min at 4 °C. Pellets were resuspended in 35% Percoll (Cytiva, Cat# 17-0891-02) and carefully overlaid onto 5 mL of 70% Percoll in 15 mL conical tubes. Layering was performed slowly to maintain distinct gradient interfaces. Gradients were centrifuged at 800 × g for 25 min at 4 °C in a swinging bucket rotor with low acceleration and minimal braking. The interphase layer containing enriched microglia was carefully collected using a P1000 pipet while avoiding disruption of the layers. Cells were washed by centrifugation at 500 × g for 5 min at 4 °C, and pellets were resuspended in ice-cold HBSS with protease and phosphatase inhibitors.

### Fluorescence-Activated Cell Sorting

Cell suspensions were incubated with antimouse CD16/CD32 (1:100; Thermo Fisher, Cat# 14-0161-82) for 10 min at 4 °C to block nonspecific binding of receptors to antibody Fc regions. Cells were stained with anti-CD11b-PE (clone M1/70, 1:170; Thermo Fisher, Cat# 12-0112-82) and anti-CD45-FITC (clone 30-F11, 1:250; Thermo Fisher, Cat# 11-0451-82) for 30 min at 4 °C in the dark. Cells were washed and resuspended in PBS containing 2% FBS supplemented with protease and phosphatase inhibitors then passed through a 70 μm filter to remove aggregates prior to sorting. Single-color compensation controls were prepared to ensure accurate gating. Sorting was performed using a Cytek Aurora 3 Laser CS spectral sorter with a 100 μm nozzle (Biomedical Research Laboratory Flow Cytometry Core Facility). Microglia were identified based on forward scatter (FSC-A) and side scatter (SSC-A) properties, with doublets excluded via FSC-A vs FSC-H gating. Microglia were defined as CD11b^high^ CD45^low/intermediate^ and sorted at >95% purity directly into a PBS solution containing 2% FBS.

### Liquid-Chromatography Electrospray Ionization Mass Spectrometry

Microglia were pelleted by centrifugation (500 × g, 5 min, 4 °C) and lysed in RIPA buffer (Thermo Fisher, Cat# 89900) supplemented with protease and phosphatase inhibitors. Samples were sonicated for 30 s on ice and incubated overnight at 4 °C with gentle agitation. Lysates were centrifuged at 14,000 × g for 15 min at 4 °C, and supernatants were collected and stored at −80 °C. Total protein concentration was measured (Supplemental Table 1) and 30 μg protein per sample was precipitated with 4× volume of ice-cold acetone overnight at −20 °C. Sample preparation for mass spectrometry was performed as described.^27^ Briefly, pellets were resuspended in 8 M urea, reduced with 1 M dithiothreitol, alkylated with 0.5 M iodoacetamide, and digested with sequencing-grade trypsin (0.5 μg/μL) in 500 nM ammonium bicarbonate and incubated for 5 h at 37 °C. Samples were desalted using C-18 ZipTips (Millipore) then dried in a SpeedVac concentrator to near-dryness (30–45 min) and reconstituted in 0.1% formic acid before separation using liquid chromatography-electrospray ionization tandem mass spectrometry (LC-ESI MS/MS). Each sample was run in 3 technical replicates. Peptide samples (2 μL injection volume) were analyzed using an Orbitrap Exploris 480 mass spectrometer coupled to an EASY-nLC 1200 nanoliquid chromatography system (Thermo Fisher) within the Center for Applied Proteomics and Molecular Medicine. Peptides were separated in a PepMap RSLC C18 column (75 μm inner diameter × 15 cm length, 2 μm particle size; Thermo Fisher) at a flow rate of 300 nL/min using a linear gradient with a final composition of 80% acetonitrile and 0.1% formic acid. Mass spectrometry was conducted in data-dependent acquisition (DDA) mode. Full MS scans were acquired in the 300–1200 *m*/*z* range at a resolution of 60,000. Fragmentation of selected precursors was performed using high-energy collision dissociation with a normalized collision energy of 28%. Internal calibration was achieved using EASY-IC. Monoisotopic precursor selection and dynamic exclusion (20s) were enabled, and only ions with charge states between +2 and +4 were selected for MS/MS acquisition.

### Protein Identification and Bioinformatic Analysis

Raw LC–MS/MS data files were processed using Proteome Discoverer v3.2 (Thermo Fisher). Peptide identification was performed using the SEQUEST HT search engine against the *Mus musculus* reference proteome (UniProt, 2025_6). Search parameters included: trypsin digestion up to 3 missed cleavages; precursor mass tolerance of 10 ppm; fragment mass tolerance of 0.02 Da; carbamidomethylation of cysteine at a fixed modification; and oxidation of methionine as a variable modification. Peptide spectral matches (PSMs) were filtered at a false discovery rate (FDR) of 5%. Proteins were considered for downstream analysis if identified in at least 2 of 3 technical replicates. Averaged PSM counts were used to calculate an abundance ratio with significance established using a Welsh’s *t* test (two-tail, *P*-value <0.05).

Gene symbols of significantly altered proteins were uploaded to STRING v12 (https://string-db.org) with a confidence score of 0.9 for protein–protein interactions in the *Mus musculus* database and an FDR of 0.01. Network visualization and clustering were performed in Cytoscape v3.10.3 using the Markov Cluster Algorithm (MCL) with an inflation parameter of 3.0 to identify functional modules, with any disconnected nodes removed. Gene Ontology (GO) enrichment analysis for biological process, molecular function, and cellular component terms and KEGG enrichment was performed using STRING v12. Enrichment terms with FDR < 0.01 were considered significant. Data visualization was performed using R (v4.5.0) with ggplot2, pheatmap and eulerr. The mass spectrometry proteomics data have been deposited to the ProteomeXchange Consortium via the PRIDE partner repository with the data set identifier PXD070417.

## Results

### Isolation and Purification of Microglia from Mouse Cerebellum

To determine the effect of chronic nicotine on cerebellar microglia proteome, microglia were isolated from the cerebellum by FACS from adult male and female mice that received either nicotine (200 μg/mL) or vehicle (2% (w/v) saccharin) as the source of drinking water. A total of 36 adult mice (9 per experimental group: male vs. female; nicotine vs. vehicle control) were tested based on their consumption for 30 consecutive days (Figure 1).

**Figure 1:**
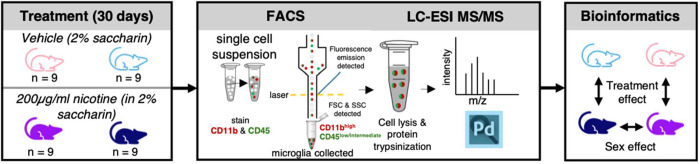
Experimental workflow for nicotine exposure and cerebellar microglia proteomics. Male and female C57BL/6J mice received nicotine (200 μg/mL in 2% saccharin) or vehicle (2% saccharin) in drinking water for 30 days. Following treatment, cerebellar tissue was dissected, dissociated, and processed by fluorescence-activated cell sorting (FACS). Microglia were isolated based on CD11b^high^ and CD45^low/intermediate^ gating followed by liquid chromatography–electrospray ionization tandem mass spectrometry (LC-ESI MS/MS) proteomic analysis to identify an effect of sex and drug treatment.

Microglia constitute approximately 10–15% of all cells within the adult rodent brain, and their density, morphology, and molecular profile can be altered in response to nicotine exposure.^8,28–30^ To characterize nicotine-induced proteomic changes in cerebellar microglia, we isolated microglia based on cell surface immunological marker proteins CD11b and CD45, which distinguish myeloid-lineage leukocytes in the CNS. Specifically, resident microglia express CD11b^high^ and CD45^low/intermediate^ while infiltrating peripheral macrophages exhibit CD11b^high^ and CD45^high^ expression.^31^ Cerebellar tissue was pooled across experimental groups from 9 mice per group then processed for FACS based on a multistep gating strategy. First, we excluded debris based on forward and side scatter properties, then identified singlets using FSC-A vs. FSC-H to eliminate doublets. From the singlet cell population, we isolated the CD11b^high^ and CD45^low/intermediate^ fraction and excluded the CD11b^high^ and CD45^high^ fraction based on evidence that these markers are present on perivascular and/or meningeal macrophages^31^ (Figure 2A–D).

**Figure 2:**
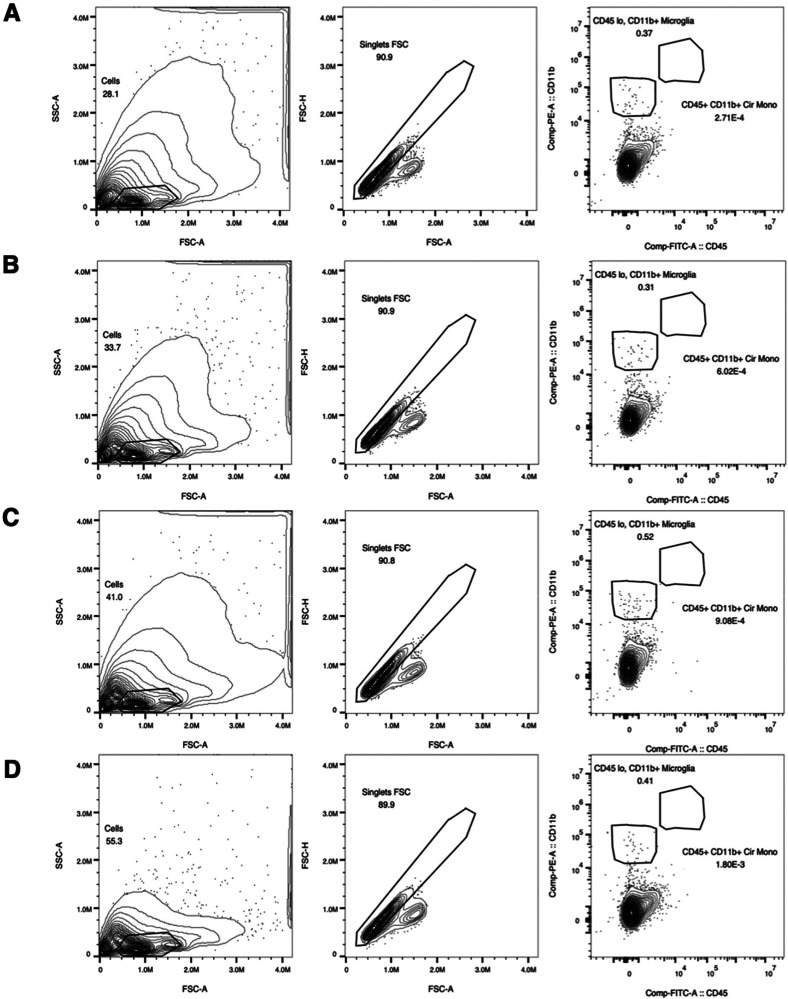
Sorting panels showing the gating strategy for microglial isolation from cerebellar tissue across the experimental groups. (A) Female control, (B) female nicotine-treated, (C) male control, and (D) male nicotine-treated. Cells were gated by size and granularity (FSC-A vs. SSC-A, left), then by exclusion of doublets (FSC-A vs. FSC-H, middle). Microglia were identified as CD11b^high^ and CD45^low/intermediate^ (boxed population, right).

Microglial (CD11b^high^ and CD45^low/intermediate^) cell yield from FACS is presented in Supplemental Table S1. We recovered approximately 40,000 microglia from female control samples (80 cells/mg tissue), 97,000 from female nicotine-treated samples (108 cells/mg tissue), 79,000 from male control samples (132 cells/mg tissue) and 72,000 from male nicotine-treated samples (90 cells/mg). Based on this result, neither sex nor nicotine treatment appeared to significantly impact microglia cell number or total protein yield. The median fluorescence intensity (MFI) of CD11b expression was similar across the samples. The CD11b^high^ CD45^low/intermediate^ cell fractions were used for subsequent proteomic analysis.

### Microglial Proteome Segregates by Sex and Nicotine Exposure

Microglia fraction, defined by CD11b^high^ and CD45^low/intermediate^ gating, was used for proteomics analysis using label-free LC-ESI MS/MS detection in DDA mode, which enables quantification of relative changes in proteins that are present across experimental samples as well as the identification of unique proteins within each sample. We performed pairwise comparisons to assess the effect of sex (male vs. female) and drug treatment (nicotine vs. vehicle control) on proteomic changes within cerebellar microglia (Figure 1).

A principal component analysis (PCA) of the microglial proteome across the four experimental conditions confirms a segregation along sex PC1 (43.9% variance) and treatment PC2 (7.8% variance). The 3 technical samples, from each experimental group, maintained high proteome relatedness within the PCA (Figure 3A). While nicotine treatment appeared to exhibit a strong impact on proteome clustering, the greatest impact within PCA was sex with male and female proteomes showing significant segregation.

**Figure 3:**
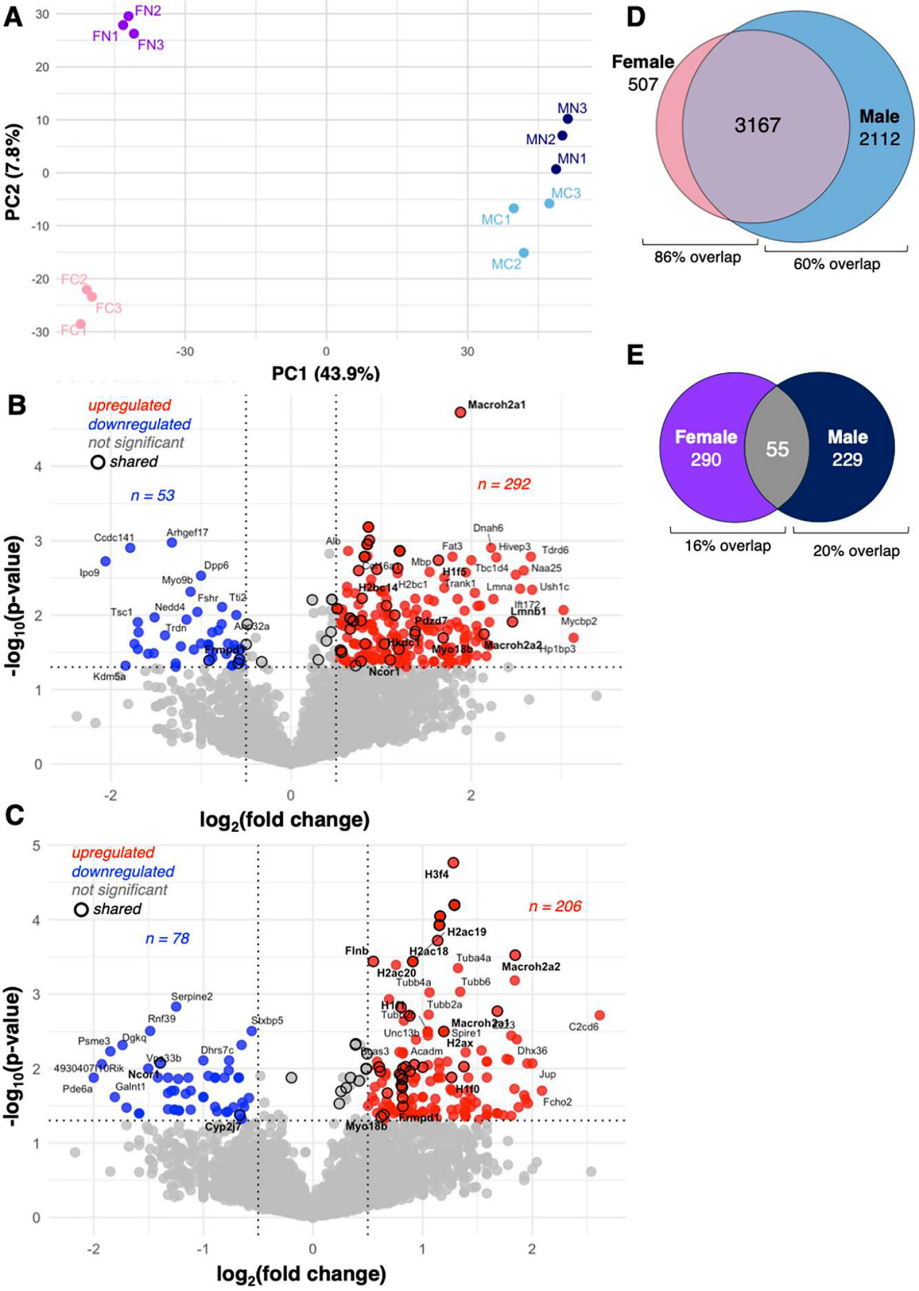
Effect of nicotine on microglial proteomes in male and female mice. (A) Principal component analysis (PCA) of cerebellar microglial proteomes showing separation by sex along PC1 (43.9% of variance) and by nicotine treatment along PC2 (7.8% of variance). FC = female control; FN = female nicotine; MC = male control; MN = male nicotine. (B) Volcano plot showing significantly altered microglial proteins in response to nicotine treatment in females. Shared proteins with males are circled. (C) Volcano plot showing significantly altered microglial proteins in response to nicotine treatment in males. Shared proteins with females are circled. (D) Venn diagram showing the overlap among all proteins in nicotine-treated female and male mice relative to sex-matched controls. (E) Venn diagram showing overlap among significantly altered proteins in nicotine-treated female and male mice relative to their sex-matched controls (*P* < 0.05).

To identify proteins that are altered in response to nicotine treatment, we compared protein abundance between nicotine-treated and control samples within each sex. In females, of the 3674 proteins shared proteins between nicotine treated and control, 345 were found to be altered based on significant changes in their abundance ratio (*P* < 0.05). Of these proteins, 292 were increased, and 53 were decreased (Figure 3B). In males, of the 5279 proteins shared between nicotine-treated and control, 285 proteins were found to be significantly altered based on changes in their abundance ratio (*P* < 0.05). Of these proteins, 206 were increased and 78 were decreased (Figure 3C). Nicotine-associated protein changes represent 9% of the total microglial proteome in females and 7% in males.

Analysis of the total proteome revealed 3167 proteins shared between female and male microglia (Figure 3D), representing 86% and 60% of female and male proteomes, respectively. Nicotine-treatment was associated with a significant change in proteome composition, with 55 proteins affected by nicotine in both sexes (Figure 3E). These shared proteins represent 16% of the female proteome previously identified to be nicotine-responsive (**circled dots** in Figure 3B) and 19% of the nicotine-responsive proteome in males (**circled dots** in Figure 3C). Although these results suggest that nicotine-induced adaptations in microglial proteomes are influenced by sex, differences in proteome coverage between males and females are descriptive. Evidence for sex-dependent nicotine responses instead emerges from the specific proteins affected, as detailed below.

### Nicotine-Mediated Alteration in Male and Female Microglia

We examined changes in the abundance of the 55 proteins that are significantly altered by chronic nicotine treatment in male and female microglia. A subset of these proteins is presented in Figure 4. Using a divergence plot that directly compares the (log_2_) fold-change in each protein between nicotine and vehicle as well as between male and female we find patterns of common (change in the same direction) and differential (change in opposite direction) responses.

**Figure 4:**
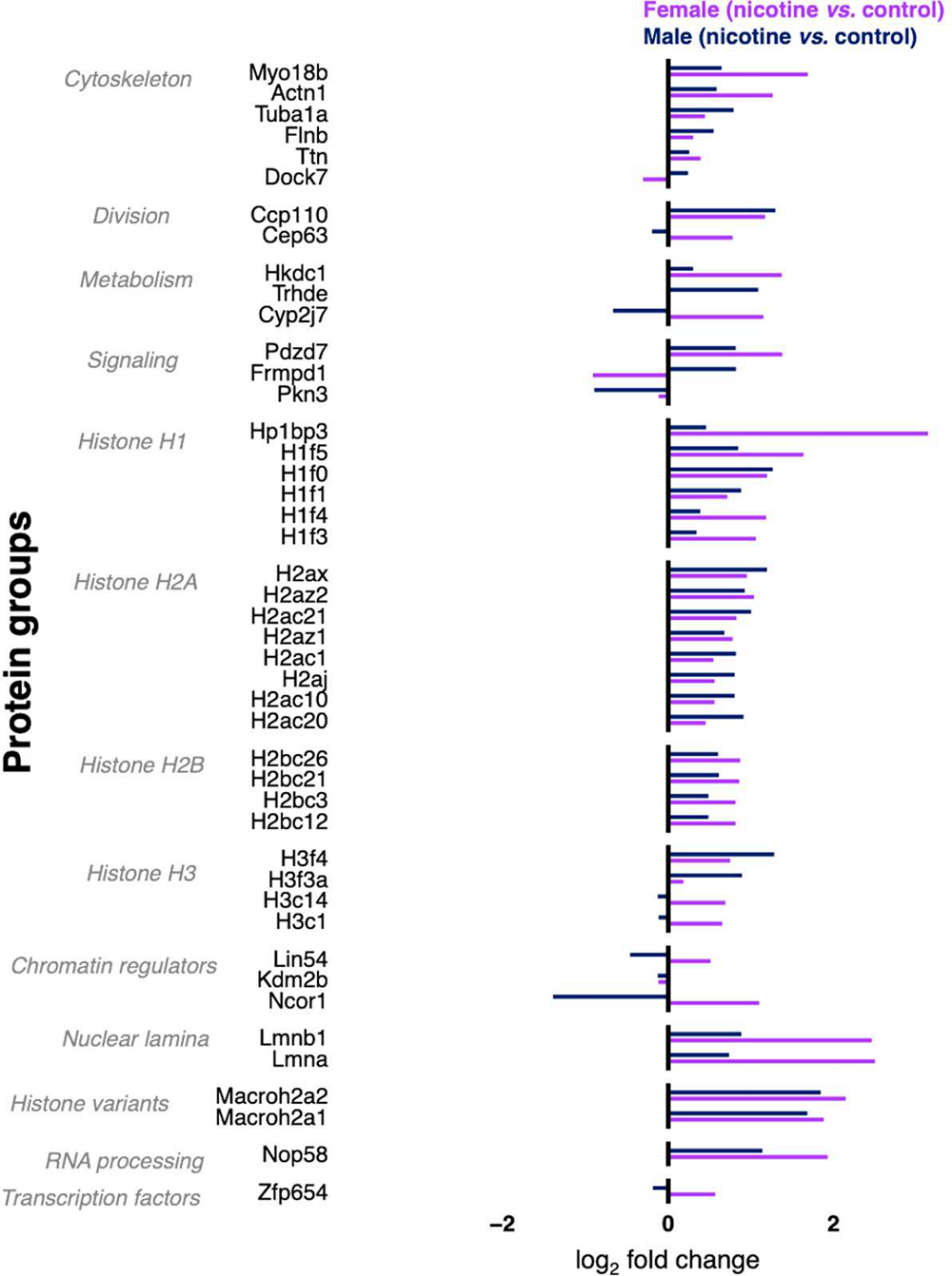
Nicotine-induced protein changes common to male and female microglia. The divergent bar plot showing log_2_ fold changes (nicotine vs. control) for proteins significantly altered in both sexes, grouped by functional category: cytoskeleton (Myo18b, Actn1, Tuba1a, Flnb, Ttn, Dock7), histones (H 1f0, H2ax, H2az1/2, H2b family, H3), nuclear lamina (Lmna, Lmnb1), metabolism (Hkdc1, Trhde), and transcription factors (Zfp654).

Most proteins showed a common change in their expression between male and female samples. This includes histones (H1f0, H2ax, H2az1/2, H2b family, H3), cytoskeletal proteins (Tuba1a, Tubb2b, Actn1, Flnb, Ttn), nuclear proteins (Lmna, Lmnb1), metabolic enzymes (Hkdc1, Trhde), and small GTPase signaling proteins (Pdzd7). While the direction of change was consistent, the magnitude of change was often much greater in females. However, several proteins exhibited sex-specific differential responses to nicotine. For example, the directionality and extent of change of chromatin regulator Ncor1 and transcriptional repressor Lin54 differed between females than males. Similarly, signaling molecules including the protein kinase Pkn3 and the PDZ domain-containing protein Frmpd1 displayed opposing directional change between the two sexes, highlighting sex-specific responses within microglia.

Gene Ontology (GO) analysis was performed to characterize the impact of nicotine and sex on microglial proteomes. Figure 5A–C shows GO terms associated with significantly altered proteins in response to nicotine relative to control within male and female microglia. Overall, the results point to enrichment terms common to males and females and highlight the effect of nicotine on proteome adaptations in histones, chromosomes, and cytoskeletal function. In females, enrichment terms for tissue development, protein localization, postsynaptic actin, and nucleosome were identified. In males, enrichment terms for ATP hydrolysis, purine binding, and presynaptic membrane were seen. To further characterize sex-specific nicotine responses, GO enrichment analysis was performed exclusively on proteins significantly altered by nicotine in females only (*n* = 290) and males only (*n* = 230), excluding the 55 proteins shared between the two sexes (Supplemental Figures S1 and S2). Overall, the GO enrichment categories identified in these sex-specific subsets are consistent with those observed in the earlier proteome analysis presented in Figure 5. These results indicate that the proteomic adaptations are predominantly driven by sex-specific responses to nicotine.

**Figure 5:**
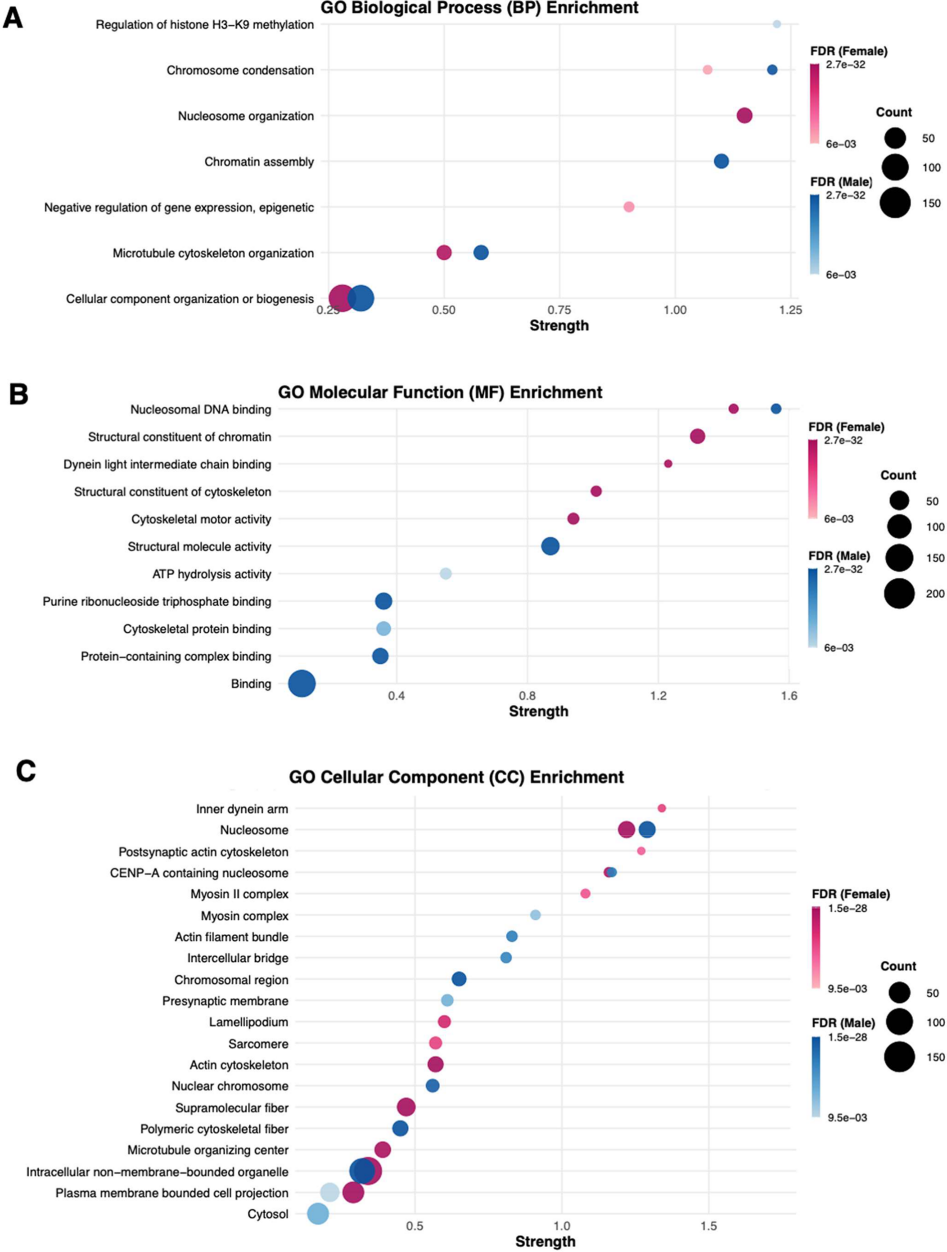
Gene ontology (GO) enrichment of differentially altered proteins in cerebellar microglia following nicotine treatment. Analysis of nicotine treated vs. control for (A) biological process (B), molecular function, and (C) cellular component. Bubble size reflects protein count; color intensity indicates the false discovery rate (FDR). Enrichment was performed using STRING’s built-in tool with a confidence interaction score of 0.9, an FDR threshold of 0.01, and a merge value ≤3 to combine related terms.

Using KEGG pathway enrichment, we explored the impact of nicotine in males and females relative to sex-matched controls. As shown in Figure 6A, KEGG enrichment indicates disease pathways including systemic lupus, alcoholism, and necroptosis within nicotine associated proteomes in males and females. Notably, male proteomes showed a greater enrichment in neurodegenerative disease terms including amyotrophic lateral sclerosis (ALS) and Huntington’s. This enrichment is consistent with evidence of greater disease risk, particularly ALS, in males who use nicotine products and suggests a role for microglial responses to nicotine use.^32,33^

**Figure 6:**
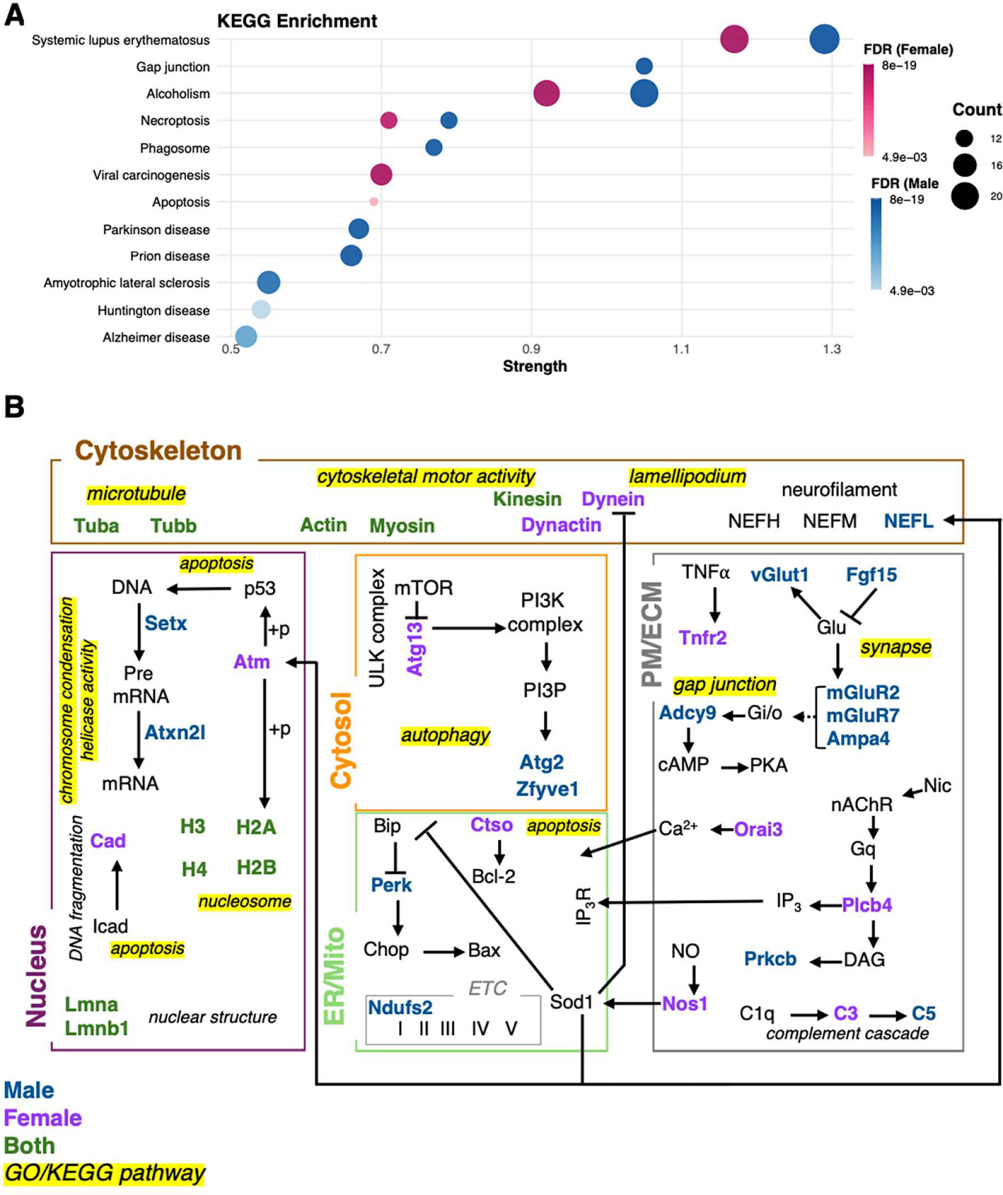
KEGG enrichment and functional organization of differentially expressed microglial proteins following nicotine exposure. (A) KEGG pathway enrichment analysis showing pathways significantly enriched in female (purple) and male (blue) microglia after nicotine treatment. Bubble size reflects protein count; color intensity indicates the sex-specific false discovery rate (FDR). Enrichment was performed using STRING’s built-in tool with a confidence interaction score of 0.9 and an FDR threshold of 0.01. (B) Schematic representation of enriched pathways from GO, KEGG, and WikiPathways (not shown), highlighting cellular compartments (nucleus, cytosol, cytoskeleton, plasma membrane/extracellular matrix (PM/ECM), and endoplasmic reticulum/mitochondria (ER/Mito)). Significantly altered proteins in males are shown in blue, in females in pink, and in both sexes in green. Pathways labeled in yellow correspond to some of the enriched terms in GO/KEGG.

To visualize the relationship among nicotine-responsive proteins, we generated an interaction model using STRING (GO and KEGG enrichment) and Wikipathways (Figure 6B). Proteins significantly altered in males are shown in blue, while proteins that are altered in females are indicated in pink and proteins altered in both sexes shown in green. The network highlights key mechanistic adaptations of signal transduction and structural pathways in microglia. Proteomic data suggests an effect of nicotine on glutamate receptor signaling in microglia through the upregulation of ionotropic alpha-amino-3-hydroxy-5-methyl-4-isoxazolepropionic acid 4 (AMPA4) and metabotropic glutamate 2 (mGluR2) protein subunits. In addition, our model suggests important changes in inflammatory signaling in microglia within females in response to nicotine through a reduction in tumor necrosis factor receptor 2 (Tnfr2) expression, diminishing their capacity for neuroprotection and synaptic maintenance.^34^ Nicotine’s effects on key cytoskeletal proteins (e.g., tubulin (Tuba/b), actin, myosin) point to a broad influence on microglial functional states (e.g., M1 or M2) that are modified by structural change as well as changes in cytoskeletal intracellular protein trafficking in males and females.

### Nicotine Enhances Chromatin, Cytoskeletal, and Metabolic Remodeling in Male Microglia

To assess sex-specific differences in microglial responses to nicotine, we directly compared the microglial response to nicotine using protein abundance between nicotine-treated males and nicotine-treated females. Heatmap visualization of Z-score normalized protein abundance revealed a consistent expression pattern within each technical replicate group (Figure 7A). This analysis involved 312 proteins that were identified to be differentially abundant between nicotine-treated males and females, demonstrating that chronic nicotine exposure produces sex-specific proteomic changes in microglia.

**Figure 7:**
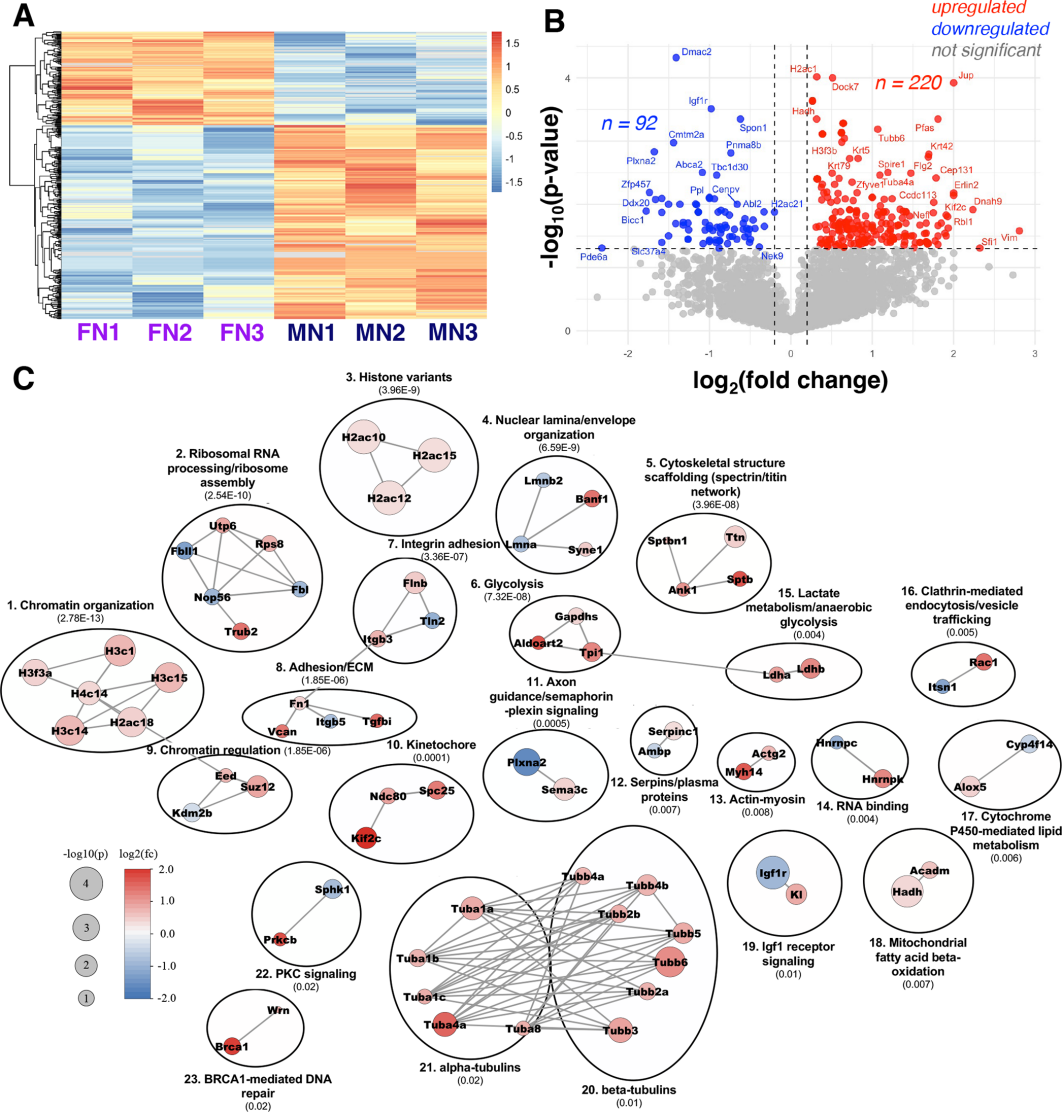
Sex-dependent microglial proteomic responses to chronic nicotine. (A) Heatmap of differentially altered proteins across nicotine-treated male (male nicotine; MN1–MN3) and female (female nicotine; FN1–FN3) replicates, showing consistent clustering by sex. (B) Volcano plot showing significantly altered proteins in males relative to females following nicotine exposure (*P* < 0.05). (C) Protein–protein interaction (PPI) network of significantly altered protein clustered using Markov Clustering (MCL) with an inflation parameter of 3, created using STRING and Cytoscape. Clusters are ordered by the PPI enrichment p-value from smallest to largest (in brackets). Node color reflects log_2_ fold change (red = upregulated in males, blue = upregulated in females); node size reflects −log_10_(*P*-value). A total of 23 significant clusters were identified including chromatin organization, PKC signaling, glycolysis, and cytoskeletal structure scaffolding.

When comparing nicotine-treated males to nicotine-treated females, 220 proteins showed significantly higher abundance in males, while 92 proteins were more abundant in females (Figure 7B). GO enrichment analysis showed that these proteomic changes are associated with cytoskeletal organization (microtubule and actin) and purine binding (ATP-related pathways) (Supplemental Figure S3). To further characterize the functional properties of proteins that are altered between nicotine-treated males and females, we performed STRING network analysis followed by clustering using Cytoscape 3.10.4. Using an MCL algorithm with an inflation parameter of 3, we identified 23 distinct protein–protein interaction (PPI) network clusters (FDR < 0.01). As shown in Figure 7C, these clusters include chromatin assembly, nucleosome positioning, rRNA processing and ribosome assembly, and histone H2A variant-mediated epigenetic regulation, and highlight nicotine’s augmented influence on transcriptional and epigenetic control in males. In addition, clusters for cell structural and morphological regulation, including nuclear lamina and envelope organization, microtubule polymerization and dynamics (α/β-tubulins), actin–myosin contractile machinery, integrin-mediated adhesion and extracellular matrix remodeling were observed. This is consistent with earlier GO enrichment findings on nicotine-mediated cytoskeletal changes and suggest enhanced cytoskeletal activity in males.

Several metabolic processes were enriched in male microglia, such as glycolytic energy metabolism, lactate metabolism, and mitochondrial fatty acid β-oxidation, indicating potential reprogramming of energy utilization that are known hallmarks of microglia activation states.^35^ Additional clusters for RNA-binding proteins and ribonucleoprotein complexes, protein kinase C (PKC) signaling, axon guidance via semaphorin–plexin signaling, IGF1R-mediated growth factor signaling, and BRCA1-dependent DNA repair were found. Interestingly, BRCA expression in microglia has been shown to support DNA repair and genomic stability, helping protect immune cells from oxidative and inflammatory stress.^36,37^ Together, these results suggest that nicotine exposure exerts a pronounced effect on male microglia by reshaping pathways central to chromatin regulation and epigenetic state, cytoskeletal and nuclear structure, and cellular metabolism, which may underlie sex-specific differences in neuroimmune activation.

To determine whether observed differences in microglial proteomes during nicotine administration reflect nicotine-induced effects rather than pre-existing (baseline) sex differences, we compared proteins that differed between male and female control (MC vs FC) and nicotine (FN vs MN) conditions. This analysis identified 374 proteins that differed between males and females in the control and 312 proteins in the nicotine conditions. We identified 43 proteins that are shared across both comparisons (Supplemental Figure S4). These 43 proteins represent less than 14% of the FN vs. MN differences, suggesting that sex-dependent proteomic differences observed following nicotine exposure are not a carryover of pre-existing sex differences, but rather proteomic adaptations to nicotine treatment. To further assess this, we examined the directionality of the 43 shared proteins to determine whether sex differences were maintained or reversed following nicotine exposure. We found that 32 proteins (∼74%) maintained the same direction, while 11 (∼26%) reversed direction following nicotine. Importantly, the 32 same-direction proteins represent only ∼10% of the total FN vs MN differences, and therefore, under the assumption that changes in all 32 proteins are entirely explained by pre-existing sex differences, approximately 90% of the sex-dependent proteomic differences following nicotine exposure remain attributable to nicotine. Taken together, these results indicate that sex-dependent proteomic differences following nicotine exposure are primarily driven by nicotine rather than baseline differences between male and female microglia.

### Enrichment of Disease Associated Proteins in Microglia

Nicotine use is an important risk factor for various human disease including inflammatory signaling that contributes to neurodegenerative conditions.^7^ Our earlier KEGG analysis points to an enrichment of neuroimmune activation within microglia from nicotine treated mice in both males and females when compared to their sex-matched controls (Figure 6A). We performed KEGG pathway enrichment by directly comparing nicotine-treated males with nicotine-treated females. As shown in Figure 8A, microglia from nicotine treated male mice showed an enrichment in proteins associated with ALS and Huntington’s relative to females.

**Figure 8:**
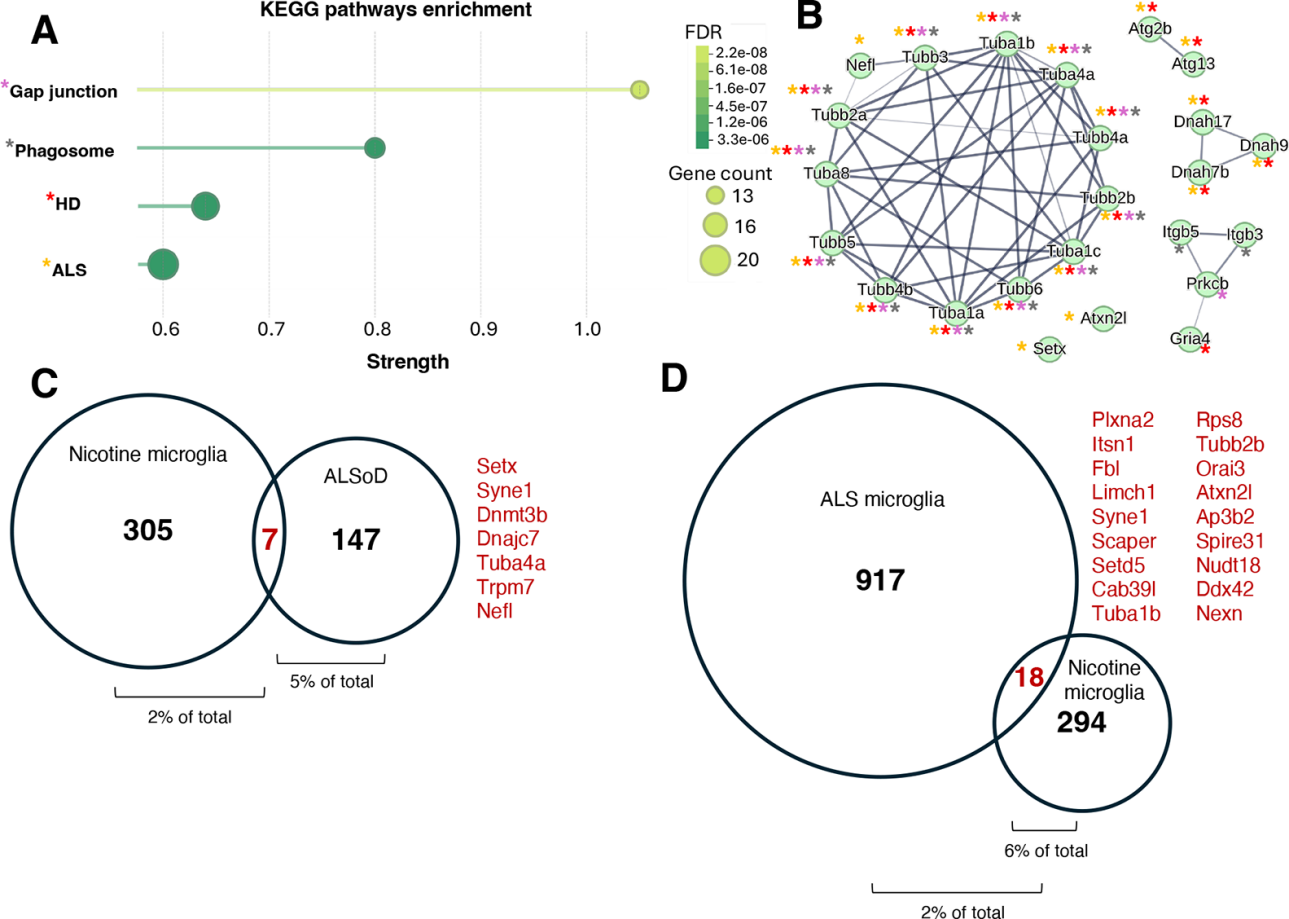
Nicotine and ALS-associated microglial proteomes. (A) KEGG pathway enrichment analysis of proteins significantly altered in nicotine-treated male relative to female microglia. Bubble size reflects protein count and color indicates the false discovery rate (FDR). Enrichment was performed using STRING’s built-in tool with a confidence interaction score of 0.9 and an FDR threshold of 0.01. (B) Protein–protein interaction (PPI) network showing connectivity among KEGG-enriched proteins. Asterisks indicate pathway associations (gap junction, purple; phagosome, green; Huntington’s disease (HD), red; Amyotrophic Lateral Sclerosis (ALS), yellow). (C) Venn diagram showing the overlap between significantly altered nicotine-responsive proteins (male microglia relative to female) and the ALS database (ALSoD). Seven overlapping proteins were identified (shown in red). (D) Venn diagram showing the overlap between significantly altered nicotine-responsive proteins (male microglia relative to female) and microglia transcriptome data from an ALS mouse model.^39^ Eighteen overlapping proteins were identified (shown in red).

STRING analysis of KEGG proteins indicates an enrichment of protein modules for tubulin, dynein, ubiquitin autophagy as well as integrin, PKC, and AMPA receptor signaling (Figure 8B). We assessed for overlap between male proteins that are significantly impacted by nicotine (relative to female) and a comprehensive ALS Online Database (ALSoD), which contains gene variants and proteins implicated in ALS.^38^ As shown in Figure 8C, we identified 7 proteins within our data set and the ALSoD. These proteins represent 5% of the ALSoD and 2% of the male microglia proteome. As expected, most of these proteins are components of the KEGG enriched ALS pathway. This includes Setx (senataxin, involved in DNA/RNA metabolism), Syne1 (nesprin-1, nuclear envelope protein), Dnmt3b (DNA methyltransferase 3 beta), Tuba4a (tubulin alpha-4A), Trpm7 (transient receptor potential cation channel) and Nefl (neurofilament light chain).

To further test these findings, we compared nicotine-induced proteomic change (in males relative to females) to transcriptomic data from microglia isolated from the spinal cord of SOD1^G93A^ mice, a well-established model of ALS.^39^ Our comparison shows that 18 proteins identified within our nicotine associated microglia proteome are detected within the ALS microglia (Figure 8D). These 18 proteins include cytoskeletal (e.g., Tubb2b), RNA processing (e.g., Rps8 and Ddx42), and signaling pathways (e.g., Plxna2) regulators that are important for disease progression.

## Discussion

In this study, we investigated the sex-specific effects of chronic nicotine on cerebellar microglial proteome using mass spectrometry. Recent studies have shown that sex differences contribute to nicotine addiction in mice.^40^ Nicotine, the primary addictive component of tobacco products including e-cigarettes, exerts widespread effects on the body through the activation of nAChRs. Multiple nAChR subtypes are expressed on microglia, including α7 and α4β2 subunits, which can modulate inflammatory processes including cytokine production and phagocytic activity through metabotropic immune signaling.^4,7,8,41,42^ While nicotine’s anti-inflammatory effects in peripheral macrophages, through α7 nAChR activation of the cholinergic anti-inflammatory pathway have been studied, the consequences of chronic nicotine exposure in microglia remains less characterized.^43–45^

### Sex-Specific Organization of the Microglial Proteome

Evidence indicates that microglia contribute to drug addiction and withdrawal through altered neuroimmune signaling,S and that drugs of abuse, including nicotine, may act directly on microglia.^46^ In addition, microglia show sex-specific differences across brain regions involved in addiction, including the nucleus accumbens, ventral tegmental area, and cerebellum.^23,47,48^ The cerebellum, beyond its classical motor functions, contributes to drug-associated learning, reward processing and reinforcement behaviors.^20^ We investigated how chronic nicotine exposure impacts male and female cerebellar microglial proteomes. Our proteomic analysis was used to identify the effect of nicotine based on two types of comparisons: (1) within-sex comparisons of nicotine-treated vs. vehicle-treated controls (2) between-sex comparisons of nicotine-treated males vs. females. Our protemic findings suggest widespread cellular adaptations to nicotine as summarized in Figure 9.

**Figure 9:**
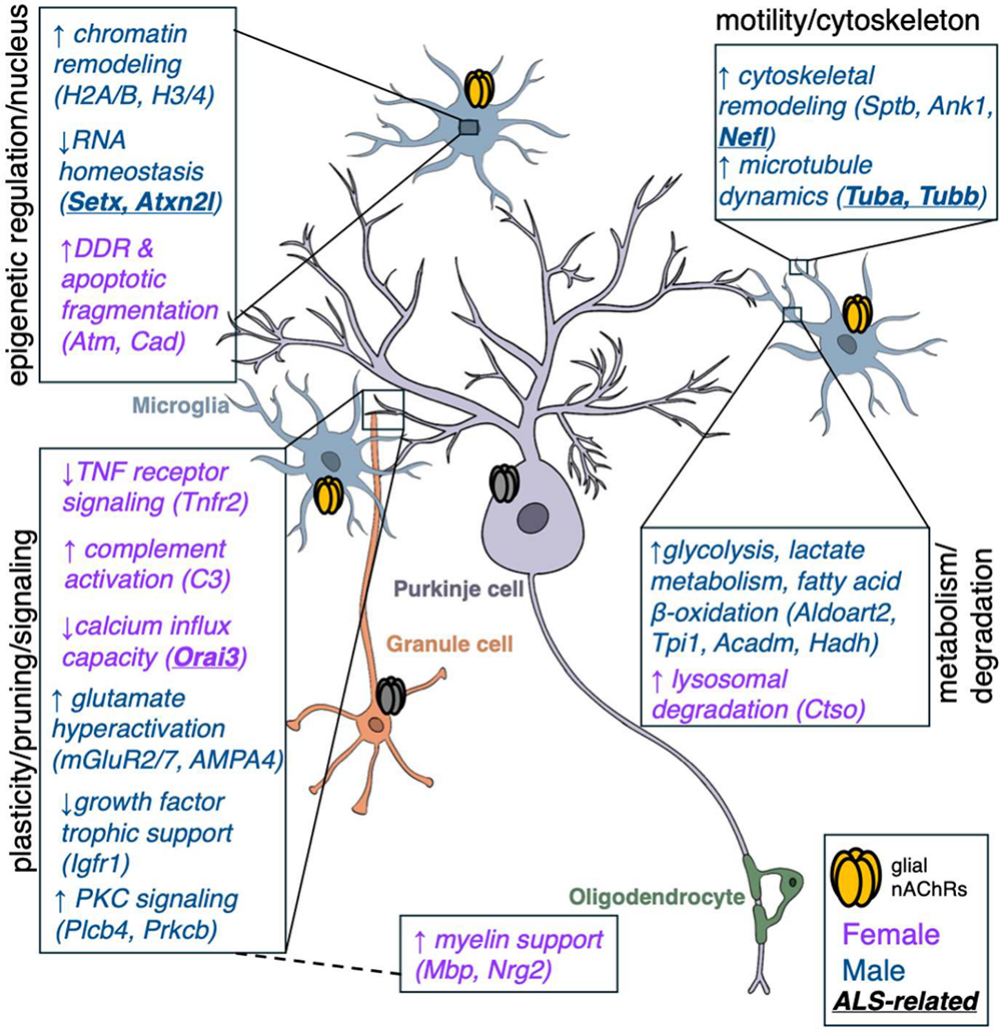
Schematic summary of sex-specific proteomic changes in microglia in response to nicotine. Illustration showing the hypothesized effect of nicotine on cerebellar synaptic activity through microglia-neuron interaction. Our proteomic analysis reveals sex-dependent differences (color-coded) in nicotine-mediated cerebellar function, as well as nicotine-induced alterations in ALS-related proteins (underlined) in microglia.

In the first comparison, our data shows that nicotine significantly alters 55 proteins that are common to microglia of males and females. Although shared, the extent and directionality of their change was not consistent between males and females. These specific proteins include chromatin regulators (Ncor1, Kdm2b, Lin54), signaling proteins (Pkn3, Frmpd1), structural regulators (Cep63, Dock7), and transcriptional regulators (Zfp654). Several histone variants and laminins are similarly upregulated in males and females by nicotine treatment and appear to be independent of sex.

Complement pathway proteins, including the C3a receptor, which mediates inflammatory signaling through the cleavage product of C3, play a critical role in synaptic regulation and the phagocytic activity of microglia.^49^ Relative to sex-matched controls, nicotine treated female mice were found to display a more pronounced change in complement C3, nitric oxide synthase Nos1 and Tnfr2 as well as myelin-associated proteins, myelin basic protein (Mbp) and neuregulin 2 (Nrg2). Microglia in females also showed proteomic signatures of DNA damage consistent with changes in (1) the expression the DNA regulator ataxia-telangiactasia mutated (Atm) protein, which is known to bind and phosphorylate p53, activating and stabilizing responses following genomic stress; and (2) an increase in caspase-activated deoxyribonuclease (Cad) and cathepsin O (Ctso).^50,51^ Cad, activated downstream of caspase-3, executes DNA cleavage and chromatin condensation during apoptosis, while Ctso is a member of the cathepsin family, which have been implicated in lysosomal proteolysis and may modulate mitochondrial integrity and BCL-2 family signaling under stress.^52–54^

Atm activity in microglia regulates immune tone and phagocytic function, and an increase in Atm expression may suggest a mechanism of neuroprotection against nicotine related genomic damage in females.^55^ This adaptive response is concurrent with our observation on an increase in Mbp, consistent with a shift toward white matter support and neuroprotection in females.^56^ Given that microglia interact with nonmyelinated axonal nodes, the increased Mbp expression observed in females following nicotine exposure may support a neuroprotective immunological bias frequently reported in female rodent models.^57^ Moreover, the upregulation of Ctso expression within females in response to nicotine suggests greater support for microglial clearance through enhanced lysosomal and phagocytic function. This may promote the maintenance of synaptic proteins during heightened neuronal activity while helping to meet the increased demands on glutamatergic synaptic clearance imposed by chronic nicotine exposure.^58^

Supporting this interpretation is our observation of upregulation in Orai3 protein expression within females in response to nicotine. Orai3 is a calcium channel subunit with resistance to oxidative inhibition. Increase in Orai3 may support a protection against cellular oxidative stress associated in microglia in response to chronic nicotine signaling.^59^ Thus, although individual nAChR subunits were not detected within our proteomic analysis, possibly due to their low relative abundance within the sample, our results support an effect of chronic nicotine on microglial calcium signaling within females. Together with Cad and complement C3 upregulation, and Ctso protein augmentation, our proteomic data suggests a strategy of neuroimmune regulation involving synaptic protein clearance and axonal support within females. However, excessive cathepsin activity can also drive neurodegenerative cascades, suggesting a potential double-edged response to chronic nicotine in female microglia.^58^ Whether the magnitude of Ctso upregulation observed here tips the balance toward neuroprotection or neurodegeneration, and how this may relate to changes in nAChR expression and function are important questions for future investigation.

### Nicotine-Driven Remodeling of Microglial Proteomic Pathways

Our second major comparison tested for the effect nicotine between male and female derived microglia. Typically, the cerebellum is characterized by a uniquely low glycolytic rate, with studies showing that males have higher susceptibility to metabolic damage contributing to neuroinflammation.^23,60^ Interestingly, cerebellar microglia exhibit distinct immunometabolic phenotypes, relying on antioxidant and lipid metabolism pathways.^23,60^ Our results suggest that nicotine may impact microglia metabolic states, where increased expression of glycolytic and lactate-related enzymes (Ldha, Ldhb, Gapdhs, Aldoart2, Tpi1) as well as altered expression of lipid metabolism regulators (Alox5; higher, Cyp4f14; lower), are observed to be more impacted in males following nicotine treatment. In response to nicotine, male derived microglia also showed an increase in mitochondrial β-oxidation enzymes (Hadh, Acadm), relative to their nicotine-treated female counterparts, suggesting a compensatory effect of nicotine on mitochondrial resilience and bioenergetics in male smokers.^61^

In addition, proteomic changes in male derived cerebellar microglia point to cytoskeletal remodeling through upregulation of spectrins (Sptbn1, Sptb), ankyrin (Ank1), and filamin (Flnb), as well as ECM regulators (Itgb3, Fn1, Vcan, Tgfbi), alongside downregulation of talin (Tln2) and integrin (Itgb5) in comparison to nicotine-treated female mice. Spectrin/ankyrin networks along with filamin proteins link the actin cortex to membranes contributing to microglial motility and surveillance behaviors.^62^ These protein changes, in males, are consistent with enhanced structural plasticity, cell-matrix interaction, and the potential for altered migratory sensing in cerebellar microglia under chronic drug use.^63^ This notion is supported by earlier studies that show that male microglia exhibit higher motility and migratory potential with greater responsiveness to inflammatory and metabolic cues.^64,65^

Pathways related to trophic signaling, cytoskeletal remodeling, and axon guidance were also altered in males, with Igf1r downregulated and Kl upregulated, suggesting potential disruption of growth factor-dependent neurotrophic support for Purkinje and granule cells.^23^ In males, we also observed an enrichment of PKC signaling (Prkcb; higher, Sphk1; lower) and changes in axon guidance regulators (Plxna2; lower, Sema3c; higher) compared to females, during chronic nicotine use. These microglial processes may impact the function of Purkinje cells and refining climbing fiber inputs, biasing toward greater cerebellar impact in males in the presence of nicotine.^23^

### Male-Specific Microglial Signatures of Neurodegenerative Vulnerability to Nicotine

Our proteomic results and KEGG analysis points to an enrichment of pathways associated with ALS and Huntington’s disease, that is greater in male mice. Nicotine-impacted microglial proteomes contain key markers of ALS with 7 proteins within the ALSoD that were altered in male mice in response to nicotine when compared to female mice.^38^ These proteins are indicated in Figure 8. Smoking is a behavioral and environmental risk factor for ALS, and epidemiological studies consistently report a higher incidence of disease in men who smoke cigarettes and other tobacco products.^32,33,66^

A downregulation of Setx and Atxn2l (ataxin 2-like), two RNA regulatory proteins of potential relevance to ALS, may compromise microglial genomic and transcriptomic stability under nicotine-induced stress. Setx is critical for resolving transcription-associated R-loops, where loss-of-function mutations result in juvenile ALS, while Atxn2l is a paralog of Atxn2, an established ALS risk gene that shares roles in RNA binding and stress granule biology, and may be recruited into Atxn2 aggregates.^67,68^ This observation aligns with our enrichment analysis showing ALS- and Huntington’s disease-related protein networks in males.

Microglia influence cerebellar glutamatergic transmission by modulating granule cell inputs onto Purkinje cells and shaping excitatory synaptic signaling.^23^ We found that microglia obtained from nicotine-treated male mice exhibited an upregulation of AMPA4 and mGluR2, and downregulation in mGluR7 receptor subunits. AMPA receptors enable microglia to sense extracellular glutamate and influence their motility and cytokine release, while mGluRs are involved in fine-tuning inflammatory responses.^69,70^ The observed receptor pattern changes suggest augmented glutamatergic responsiveness, possibly due to greater excitatory transmission, and a shift to pro-inflammatory signaling in males under chronic nicotine. This male-specific response contrasts with the protective adaptations observed in females, where mechanisms such as reduced oxidative sensitivity and dampened microglial activation appear to buffer against chronic nicotine-induced cell damage.

## Conclusions

Our findings suggest that nicotine exposure results in sex-dependent proteomic adaptations in cerebellar microglia, reshaping pathways involved in metabolism, immune signaling, and synaptic communication. Proteomic shifts in males suggest that nicotine biases cerebellar microglia toward a state that is associated with increased neurodegenerative disease risk, while in females, nicotine can mitigate genomic and repair functions. Microglia responses to drugs of abuse may remodel the circuitry of the cerebellum including climbing fiber and parallel fiber synapses onto Purkinje cells. These pathways encode repetitive motor sequences and underlie cerebellar-dependent learning and habit consolidation.^23–25,71,72^ Thus, microglial responses to nicotine can modulate procedural learning underlying smoking automaticity, motor rituals, and may a role in relapse vulnerability.^21^ Future work should extend these observations to explore the contributions of microglia in nicotine-induced changes in neuronal connectivity and behavior.

## Supplementary Material



## Notes

GO enrichment analysis of differentially expressed proteins in nicotine females relative to control females (Figure S1); GO enrichment analysis of differentially expressed proteins in nicotine males relative to control males (Figure S2); GO enrichment analysis of differentially expressed proteins in nicotine males relative to nicotine females (Figure S3); overlap between sex-dependent microglial proteome differences in control and nicotine-exposed groups (Figure S4); and summary of microglia isolation metrics by sex and treatment condition (Table S1) (PDF)

## References

[1] Letsinger A. C., Gu Z., Yakel J. L. (2022). Α7 Nicotinic Acetylcholine Receptors in the Hippocampal
Circuit: Taming Complexity. Trends in Neurosciences.

[2] Shytle R. D., Mori T., Townsend K., Vendrame M., Sun N., Zeng J., Ehrhart J., Silver A. A., Sanberg P. R., Tan J. (2004). Cholinergic Modulation of Microglial Activation by Α7 Nicotinic
Receptors. Journal of Neurochemistry.

[3] An M. C., Lin W., Yang J., Dominguez B., Padgett D., Sugiura Y., Aryal P., Gould T. W., Oppenheim R. W., Hester M. E., Kaspar B. K., Ko C.-P., Lee K.-F. (2010). Acetylcholine
Negatively Regulates Development of the Neuromuscular Junction through
Distinct Cellular Mechanisms. Proc. Natl. Acad.
Sci. U. S. A..

[4] Kalkman H. O., Feuerbach D. (2016). Modulatory
Effects of Α7 nAChRs on the Immune
System and Its Relevance for CNS Disorders. Cell. Mol. Life Sci..

[5] McKay B. E., Placzek A. N., Dani J. A. (2007). Regulation
of Synaptic Transmission
and Plasticity by Neuronal Nicotinic Acetylcholine Receptors. Biochem. Pharmacol..

[6] Mizrachi T., Vaknin-Dembinsky A., Brenner T., Treinin M. (2021). Neuroinflammation Modulation
via Α7 Nicotinic Acetylcholine Receptor and Its Chaperone, RIC-3. Molecules.

[7] Piao W.-H., Campagnolo D., Dayao C., Lukas R. J., Wu J., Shi F.-D. (2009). Nicotine and Inflammatory Neurological Disorders. Acta Pharmacol Sin.

[8] Soares A. R., Garcia-Rivas V., Fai C., Thomas M., Zheng X., Picciotto M. R., Mineur Y. S. (2025). Sex Differences in the Microglial
Response to Stress and Chronic Alcohol Exposure in Mice. Biology of Sex Differences.

[9] Cornell J., Salinas S., Huang H.-Y., Zhou M. (2022). Microglia
Regulation
of Synaptic Plasticity and Learning and Memory. Neural Regen Res..

[10] Barko K., Shelton M., Xue X., Afriyie-Agyemang Y., Puig S., Freyberg Z., Tseng G. C., Logan R. W., Seney M. L. (2022). Brain Region- and Sex-Specific Transcriptional Profiles
of Microglia. Front. Psychiatry.

[11] Bishnoi I. R., Bordt E. A. (2025). Sex and Region-Specific
Differences in Microglial Morphology
and Function Across Development. Neuroglia.

[12] Guan Y.-Z., Jin X.-D., Guan L.-X., Yan H.-C., Wang P., Gong Z., Li S.-J., Cao X., Xing Y.-L., Gao T.-M. (2015). Nicotine Inhibits Microglial Proliferation
and Is Neuroprotective
in Global Ischemia Rats. Mol. Neurobiol.

[13] Adeluyi A., Guerin L., Fisher M. L., Galloway A., Cole R. D., Chan S. S. L., Wyatt M. D., Davis S. W., Freeman L. R., Ortinski P. I., Turner J. R. (2019). Microglia Morphology and Proinflammatory
Signaling in the Nucleus Accumbens during Nicotine Withdrawal. Sci. Adv..

[14] Zhang Q., Lu Y., Bian H., Guo L., Zhu H. (2017). Activation of the Α7
Nicotinic Receptor Promotes Lipopolysaccharide-Induced Conversion
of M1Microglia to M2. Am. J. Transl Res..

[15] De
Simone R., Ajmone-Cat M. A., Carnevale D., Minghetti L. (2005). Activation of Α7 Nicotinic Acetylcholine Receptor
by Nicotine Selectively Up-Regulates Cyclooxygenase-2 and Prostaglandin
E2 in Rat Microglial Cultures. Journal of Neuroinflammation.

[16] King J. R., Gillevet T. C., Kabbani N. (2017). A G Protein-Coupled
Α7 Nicotinic
Receptor Regulates Signaling and TNF-α Release in Microglia. FEBS Open Bio.

[17] Egea J., Buendia I., Parada E., Navarro E., León R., Lopez M. G. (2015). Anti-Inflammatory
Role of Microglial Alpha7 nAChRs
and Its Role in Neuroprotection. Biochem. Pharmacol..

[18] Piovesana R., Salazar Intriago M. S., Dini L., Tata A. M. (2021). Cholinergic Modulation
of Neuroinflammation: Focus on Α7 Nicotinic Receptor. Int. J. Mol. Sci..

[19] Cai Z., Wang P., Liu B., Zou Y., Wu S., Tian J., Dan G., Ma J., Wu G., Zhang J., Huang B. (2022). To Explore the Mechanism of Tobacco
Addiction Using Structural and Functional MRI: A Preliminary Study
of the Role of the Cerebellum-Striatum Circuit. Brain Imaging Behav.

[20] Wang Y., Lan Y. (2023). The Role of the Cerebellum in Drug
Reward: A Review. JIN.

[21] Zhang W., Tian Y., Yang X., He B., Zhang H., Zhang Q., Mei Y. (2025). Arginine Metabolism
and Adenosine
Receptor Signals in the Cerebellum Contribute to Nicotine Withdrawal-Induced
Anxiety/Depression-Like Behaviours. Addiction
Biology.

[22] Stoessel M. B., Majewska A. K. (2021). Little Cells of the Little Brain: Microglia in Cerebellar
Development and Function. Trends in Neurosciences.

[23] Dukhinova M. S., Guo J., Shen E., Liu W., Huang W., Shen Y., Wang L. (2026). Cerebellar Microglia: On the Edge between Neuroinflammation and Neuroregulation. Neural Regen Res..

[24] Ayata P., Badimon A., Strasburger H. J., Duff M. K., Montgomery S. E., Loh Y.-H. E., Ebert A., Pimenova A. A., Ramirez B. R., Chan A. T., Sullivan J. M., Purushothaman I., Scarpa J. R., Goate A. M., Busslinger M., Shen L., Losic B., Schaefer A. (2018). Epigenetic Regulation
of Brain Region-Specific Microglia Clearance Activity. Nat. Neurosci.

[25] Tay T. L., Mai D., Dautzenberg J., Fernández-Klett F., Lin G., Sagar, Datta M., Drougard A., Stempfl T., Ardura-Fabregat A., Staszewski O., Margineanu A., Sporbert A., Steinmetz L. M., Pospisilik J. A., Jung S., Priller J., Grün D., Ronneberger O., Prinz M. (2017). A New Fate Mapping System Reveals
Context-Dependent Random or Clonal Expansion of Microglia. Nat. Neurosci.

[26] Jung Y., Hsieh L. S., Lee A. M., Zhou Z., Coman D., Heath C. J., Hyder F., Mineur Y. S., Yuan Q., Goldman D., Bordey A., Picciotto M. R. (2016). An Epigenetic
Mechanism Mediates Developmental Nicotine Effects on Neuronal Structure
and Behavior. Nat. Neurosci.

[27] Graur A., Erickson N., Sinclair P., Nusir A., Kabbani N. (2025). HIV-1 Gp120
Interactions with Nicotine Modulate Mitochondrial Network Properties
and Amyloid Release in Microglia. Neurochem.
Res..

[28] Frost J. L., Schafer D. P. (2016). Microglia: Architects
of the Developing Nervous System. Trends Cell
Biol..

[29] Lawson L. J., Perry V. H., Dri P., Gordon S. (1990). Heterogeneity
in the
Distribution and Morphology of Microglia in the Normal Adult Mouse
Brain. Neuroscience.

[30] Vilca S. J., Margetts A. V., Pollock T. A., Tuesta L. M. (2023). Transcriptional
and Epigenetic Regulation of Microglia in Substance Use Disorders. Molecular and Cellular Neuroscience.

[31] Martin E., El-Behi M., Fontaine B., Delarasse C. (2017). Analysis of
Microglia and Monocyte-Derived Macrophages from the Central Nervous
System by Flow Cytometry. J. Vis. Exp..

[32] Manjaly Z. R., Scott K. M., Abhinav K., Wijesekera L., Ganesalingam J., Goldstein L. H., Janssen A., Dougherty A., Willey E., Stanton B. R., Turner M. R., Ampong M.-A., Sakel M., Orrell R. W., Howard R., Shaw C. E., Leigh P. N., Al-Chalabi A. (2010). The Sex Ratio in Amyotrophic Lateral
Sclerosis: A Population Based Study. Amyotrophic
Lateral Sclerosis.

[33] Wang H., O’Reilly É.
J., Weisskopf M. G., Logroscino G., McCullough M. L., Thun M., Schatzkin A., Kolonel L. N., Ascherio A. (2011). Smoking and Risk of Amyotrophic Lateral
Sclerosis: A Pooled Analysis of Five Prospective Cohorts. Arch Neurol.

[34] Raffaele S., Thougaard E., Laursen C. C. H., Gao H., Andersen K. M., Nielsen P. V., Ortí-Casañ N., Blichfeldt-Eckhardt M., Koch S., Deb-Chatterji M., Magnus T., Stubbe J., Madsen K., Meyer M., Degn M., Eisel U. L. M., Wlodarczyk A., Fumagalli M., Clausen B. H., Brambilla R., Lambertsen K. L. (2024). Microglial TNFR2 Signaling Regulates the Inflammatory
Response after CNS Injury in a Sex-Specific Fashion. Brain Behav Immun.

[35] Miao J., Chen L., Pan X., Li L., Zhao B., Lan J. (2023). Microglial Metabolic Reprogramming:
Emerging Insights and Therapeutic
Strategies in Neurodegenerative Diseases. Cell
Mol. Neurobiol.

[36] Leung E., Hazrati L.-N. (2021). Breast Cancer Type
1 and Neurodegeneration: Consequences
of Deficient DNA Repair. Brain Commun..

[37] Noristani H. N., Gerber Y. N., Sabourin J.-C., Le Corre M., Lonjon N., Mestre-Frances N., Hirbec H. E., Perrin F. E. (2017). RNA-Seq Analysis
of Microglia Reveals Time-Dependent Activation of Specific Genetic
Programs Following Spinal Cord Injury. Front.
Mol. Neurosci..

[38] ALSoD https://alsod.ac.uk/ (accessed 2025-10-07).

[39] Chiu I. M., Morimoto E. T. A., Goodarzi H., Liao J. T., O’Keeffe S., Phatnani H. P., Muratet M., Carroll M. C., Levy S., Tavazoie S., Myers R. M., Maniatis T. (2013). A Neurodegeneration-Specific
Gene-Expression Signature of Acutely Isolated Microglia from an Amyotrophic
Lateral Sclerosis Mouse Model. Cell Reports.

[40] Lee A. M., Mansuri M. S., Wilson R. S., Lam T. T., Nairn A. C., Picciotto M. R. (2021). Sex Differences
in the Ventral Tegmental Area and Nucleus
Accumbens Proteome at Baseline and Following Nicotine Exposure. Front. Mol. Neurosci..

[41] Takata K., Kimura H., Yanagisawa D., Harada K., Nishimura K., Kitamura Y., Shimohama S., Tooyama I. (2022). Nicotinic Acetylcholine
Receptors and Microglia as Therapeutic and Imaging Targets in Alzheimer’s
Disease. Molecules.

[42] Sinclair P., Kabbani N. (2023). Ionotropic and Metabotropic
Responses by Alpha 7 Nicotinic
Acetylcholine Receptors. Pharmacol. Res..

[43] Soares A. R., Picciotto M. R. (2024). Nicotinic
Regulation of Microglia: Potential Contributions
to Addiction. J. Neural Transm.

[44] Kumar M., Adeluyi A., Anderson E. L., Turner J. R. (2020). Glial Cells as Therapeutic
Targets for Smoking Cessation. Neuropharmacology.

[45] Pavlov V. A., Wang H., Czura C. J., Friedman S. G., Tracey K. J. (2003). The Cholinergic
Anti-Inflammatory Pathway: A Missing Link in Neuroimmunomodulation. Mol. Med..

[46] Lacagnina M. J., Rivera P. D., Bilbo S. D. (2017). Glial and
Neuroimmune Mechanisms
as Critical Modulators of Drug Use and Abuse. Neuropsychopharmacol.

[47] Becker J. B., Chartoff E. (2019). Sex Differences in
Neural Mechanisms Mediating Reward
and Addiction. Neuropsychopharmacology.

[48] Kopec A. M., Smith C. J., Ayre N. R., Sweat S. C., Bilbo S. D. (2018). Microglial
Dopamine Receptor Elimination Defines Sex-Specific Nucleus Accumbens
Development and Social Behavior in Adolescent Rats. Nat. Commun..

[49] Young K. G., Yan K., Picketts D. J. (2019). C3aR Signaling and
Gliosis in Response to Neurodevelopmental
Damage in the Cerebellum. J. Neuroinflammation.

[50] Banin S., Moyal L., Shieh S.-Y., Taya Y., Anderson C. W., Chessa L., Smorodinsky N. I., Prives C., Reiss Y., Shiloh Y., Ziv Y. (1998). Enhanced Phosphorylation
of P53 by
ATM in Response to DNA Damage. Science.

[51] Cheng Q., Chen J. (2010). Mechanism of P53 Stabilization by ATM after DNA Damage. Cell Cycle.

[52] Soond S. M., Kozhevnikova M. V., Savvateeva L. V., Townsend P. A., Zamyatnin A. A. (2021). Intrinsically
Connected: Therapeutically Targeting the Cathepsin Proteases and the
Bcl-2 Family of Protein Substrates as Co-Regulators of Apoptosis. International Journal of Molecular Sciences.

[53] Larsen B. D., So̷rensen C. S. (2017). The Caspase-Activated
DNase: Apoptosis and Beyond. FEBS J..

[54] Moeed A., Thilmany N., Beck F., Puthussery B. K., Ortmann N., Haimovici A., Badr M. T., Haghighi E. B., Boerries M., Öllinger R., Rad R., Kirschnek S., Gentle I. E., Donakonda S., Petric P. P., Hummel J. F., Pfaffendorf E., Zanetta P., Schell C., Schwemmle M., Weber A., Häcker G. (2024). The Caspase-Activated DNase Drives
Inflammation and Contributes to Defense against Viral Infection. Cell Death Differ..

[55] Bourseguin J., Cheng W., Talbot E., Hardy L., Lai J., Jeffries A. M., Lodato M. A., Lee E. A., Khoronenkova S. V. (2022). Persistent
DNA Damage Associated with ATM Kinase Deficiency Promotes Microglial
Dysfunction. Nucleic Acids Res..

[56] McNamara N. B., Munro D. A. D., Bestard-Cuche N., Uyeda A., Bogie J. F. J., Hoffmann A., Holloway R. K., Molina-Gonzalez I., Askew K. E., Mitchell S., Mungall W., Dodds M., Dittmayer C., Moss J., Rose J., Szymkowiak S., Amann L., McColl B. W., Prinz M., Spires-Jones T. L., Stenzel W., Horsburgh K., Hendriks J. J. A., Pridans C., Muramatsu R., Williams A., Priller J., Miron V. E. (2023). Microglia
Regulate Central Nervous System Myelin Growth and Integrity. Nature.

[57] Ronzano R., Roux T., Thetiot M., Aigrot M. S., Richard L., Lejeune F. X., Mazuir E., Vallat J. M., Lubetzki C., Desmazières A. (2021). Microglia-Neuron
Interaction at Nodes of Ranvier Depends
on Neuronal Activity through Potassium Release and Contributes to
Remyelination. Nat. Commun..

[58] Pišlar A., Bolčina L., Kos J. (2021). New Insights into the
Role of Cysteine
Cathepsins in Neuroinflammation. Biomolecules.

[59] Zhang X., Xin P., Yoast R. E., Emrich S. M., Johnson M. T., Pathak T., Benson J. C., Azimi I., Gill D. L., Monteith G. R., Trebak M. (2020). Distinct Pharmacological
Profiles of ORAI1, ORAI2,
and ORAI3 Channels. Cell Calcium.

[60] Vela J. M., Dalmau I., González B., Castellano B. (1995). Morphology
and Distribution of Microglial Cells in the Young and Adult Mouse
Cerebellum. Journal of Comparative Neurology.

[61] Malińska D., Więckowski M. R., Michalska B., Drabik K., Prill M., Patalas-Krawczyk P., Walczak J., Szymański J., Mathis C., Van der Toorn M., Luettich K., Hoeng J., Peitsch M. C., Duszyński J., Szczepanowska J. (2019). Mitochondria
as a Possible Target for Nicotine Action. J.
Bioenerg Biomembr.

[62] Franco-Bocanegra D. K., McAuley C., Nicoll J. A. R., Boche D. (2019). Molecular
Mechanisms
of Microglial Motility: Changes in Ageing and Alzheimer’s Disease. Cells.

[63] Nguyen P. T., Dorman L. C., Pan S., Vainchtein I. D., Han R. T., Nakao-Inoue H., Taloma S. E., Barron J. J., Molofsky A. B., Kheirbek M. A., Molofsky A. V. (2020). Microglial Remodeling
of the Extracellular Matrix Promotes Synapse Plasticity. Cell.

[64] Boghozian R., Sharma S., Narayana K., Cheema M., Brown C. E. (2023). Sex and
Interferon Gamma Signaling Regulate Microglia Migration in the Adult
Mouse Cortex in Vivo. Proc. Natl. Acad. Sci.
U. S. A..

[65] Guneykaya D., Ivanov A., Hernandez D. P., Haage V., Wojtas B., Meyer N., Maricos M., Jordan P., Buonfiglioli A., Gielniewski B., Ochocka N., Cömert C., Friedrich C., Artiles L. S., Kaminska B., Mertins P., Beule D., Kettenmann H., Wolf S. A. (2018). Transcriptional
and Translational Differences of Microglia from Male and Female Brains. Cell Reports.

[66] Kim, K. ; Ko, D. S. ; Kim, J.-W. ; Lee, D. ; Son, E. ; Kim, H.-W. ; Song, T.-J. ; Kim, Y. H. Association of Smoking with Amyotrophic Lateral Sclerosis: A Systematic Review, Meta-Analysis, and Dose-Response Analysis. Tob Induc Dis 2024, 22, 1 10.18332/tid/175731. PMC10795623 38239315

[67] Grunseich C., Wang I. X., Watts J. A., Burdick J. T., Guber R. D., Zhu Z., Bruzel A., Lanman T., Chen K., Schindler A. B., Edwards N., Ray-Chaudhury A., Yao J., Lehky T., Piszczek G., Crain B., Fischbeck K. H., Cheung V. G. (2018). Senataxin Mutation Reveals How R-Loops Promote Transcription
by Blocking DNA Methylation at Gene Promoters. Mol. Cell.

[68] Kaehler C., Isensee J., Nonhoff U., Terrey M., Hucho T., Lehrach H., Krobitsch S. (2012). Ataxin-2-Like Is a Regulator of Stress
Granules and Processing Bodies. PLoS One.

[69] Ceprian M., Fulton D. (2019). Glial Cell AMPA Receptors in Nervous System Health,
Injury and Disease. International Journal of
Molecular Sciences.

[70] Spampinato S. F., Costantino G., Merlo S., Canonico P. L., Sortino M. A. (2022). Microglia
Contributes to BAF-312 Effects on Blood–Brain Barrier Stability. Biomolecules.

[71] Kana V., Desland F. A., Casanova-Acebes M., Ayata P., Badimon A., Nabel E., Yamamuro K., Sneeboer M., Tan I.-L., Flanigan M. E., Rose S. A., Chang C., Leader A., Le Bourhis H., Sweet E. S., Tung N., Wroblewska A., Lavin Y., See P., Baccarini A., Ginhoux F., Chitu V., Stanley E. R., Russo S. J., Yue Z., Brown B. D., Joyner A. L., De Witte L. D., Morishita H., Schaefer A., Merad M. (2019). CSF-1 Controls Cerebellar Microglia
and Is Required for Motor Function and Social Interaction. J. Exp Med..

[72] Nakayama H., Abe M., Morimoto C., Iida T., Okabe S., Sakimura K., Hashimoto K. (2018). Microglia
Permit Climbing Fiber Elimination by Promoting
GABAergic Inhibition in the Developing Cerebellum. Nat. Commun..

