# Supplementary material for: Microglial Adaptations to Chronic Nicotine in the Cerebellum: Proteomic Evidence for Neuroimmune Vulnerability
Source: J Proteome Res. 2026 Mar 30;25(5):2359–73. doi: 10.1021/acs.jproteome.5c01027 (PMC13140143; doi:10.1021/acs.jproteome.5c01027)
Supplement: Supplementary file 1 [file pr5c01027_si_001.pdf]

Supporting information

Microglial Adaptations to Chronic Nicotine in the Cerebellum: Proteomic Evidence for Neuroimmune Vulnerability

*Aya Nusir[1], Scott M. Anthony[2], Weidong Zhou[3], Nadine Kabbani[1]**
[1]Interdisciplinary Program in Neuroscience, School of Systems Biology, George Mason University, Fairfax, VA 22030, USA
[2]Biomedical Research Laboratory, George Mason University, 10650 Pyramid Place, Manassas, VA 20110, USA
[3]Center for Applied Proteomics and Molecular Medicine, George Mason University, 10920 George Mason Circle, Manassas, VA 20110, USA
*Correspondence: nkabbani@gmu.edu

Table of contents

Supplemental Figure S1. GO enrichment analysis of differentially expressed proteins in nicotine females relative to control females.
Supplemental Figure S2. GO enrichment analysis of differentially expressed proteins in nicotine males relative to control males.
Supplemental Figure S3. GO enrichment analysis of differentially expressed proteins in nicotine males relative to nicotine females.
Supplemental Figure S4. Overlap between sex-dependent microglial proteome differences in control and nicotine-exposed groups.

Supplemental Table S1. Summary of microglia isolation metrics by sex and treatment condition.

Figure S1. GO enrichment analysis of differentially expressed proteins in nicotine females relative to control females. (A) biological process, (B) cellular component, and (C) molecular function. Bubble size reflects gene count per category; color indicates false discovery rate (FDR). GO enrichment performed using STRING's built-in tool with 0.9 confidence interaction score, FDR < 0.01 and a merge value ≤ 3 to combine related terms.

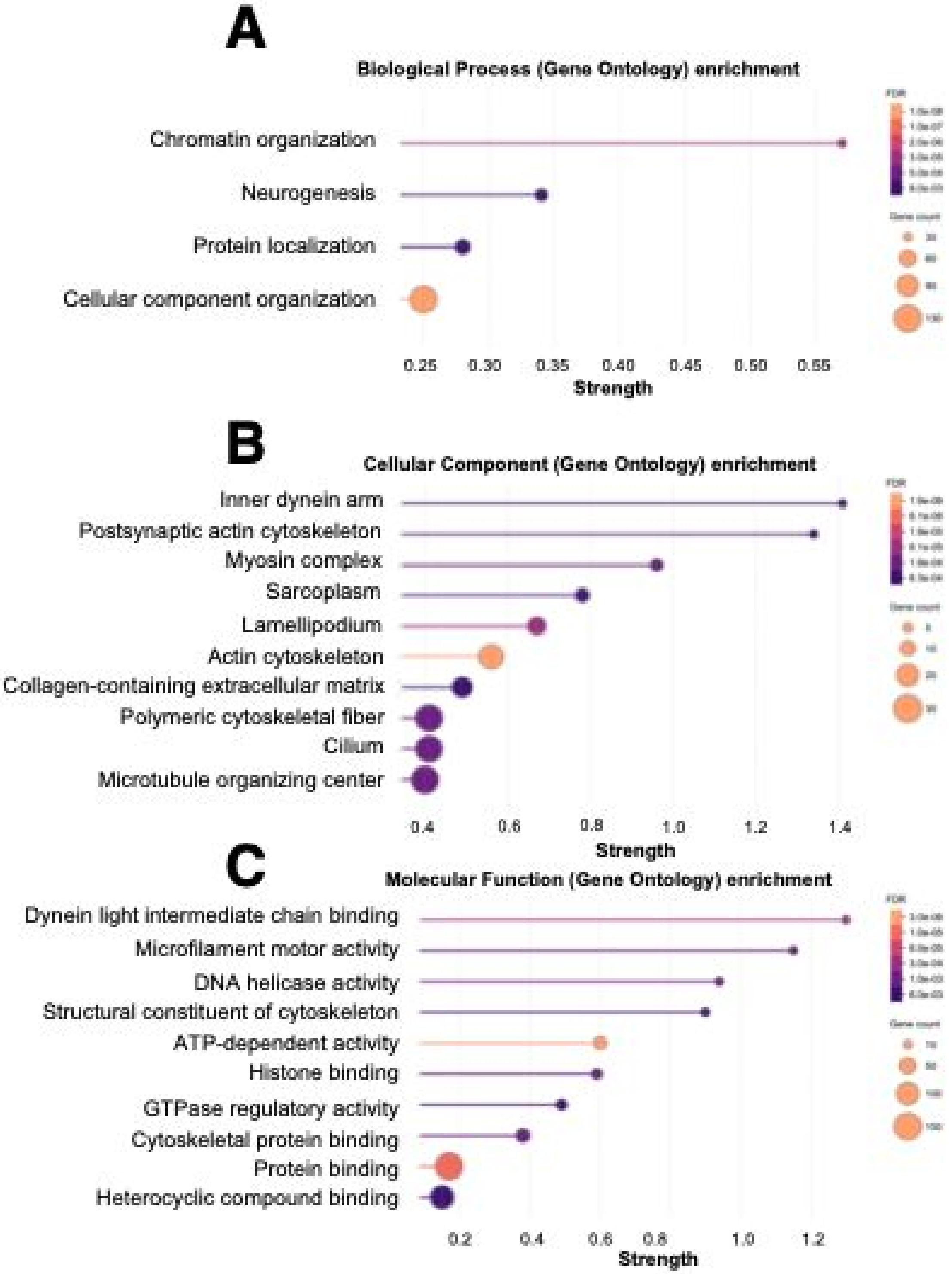

Figure S2. GO enrichment analysis of differentially expressed proteins in nicotine males relative to control males. (A) biological process, (B) cellular component, and (C) molecular function. Bubble size reflects gene count per category; color indicates false discovery rate (FDR). GO enrichment performed using STRING's built-in tool with 0.9 confidence interaction score, FDR < 0.01 and a merge value ≤ 3 to combine related terms.

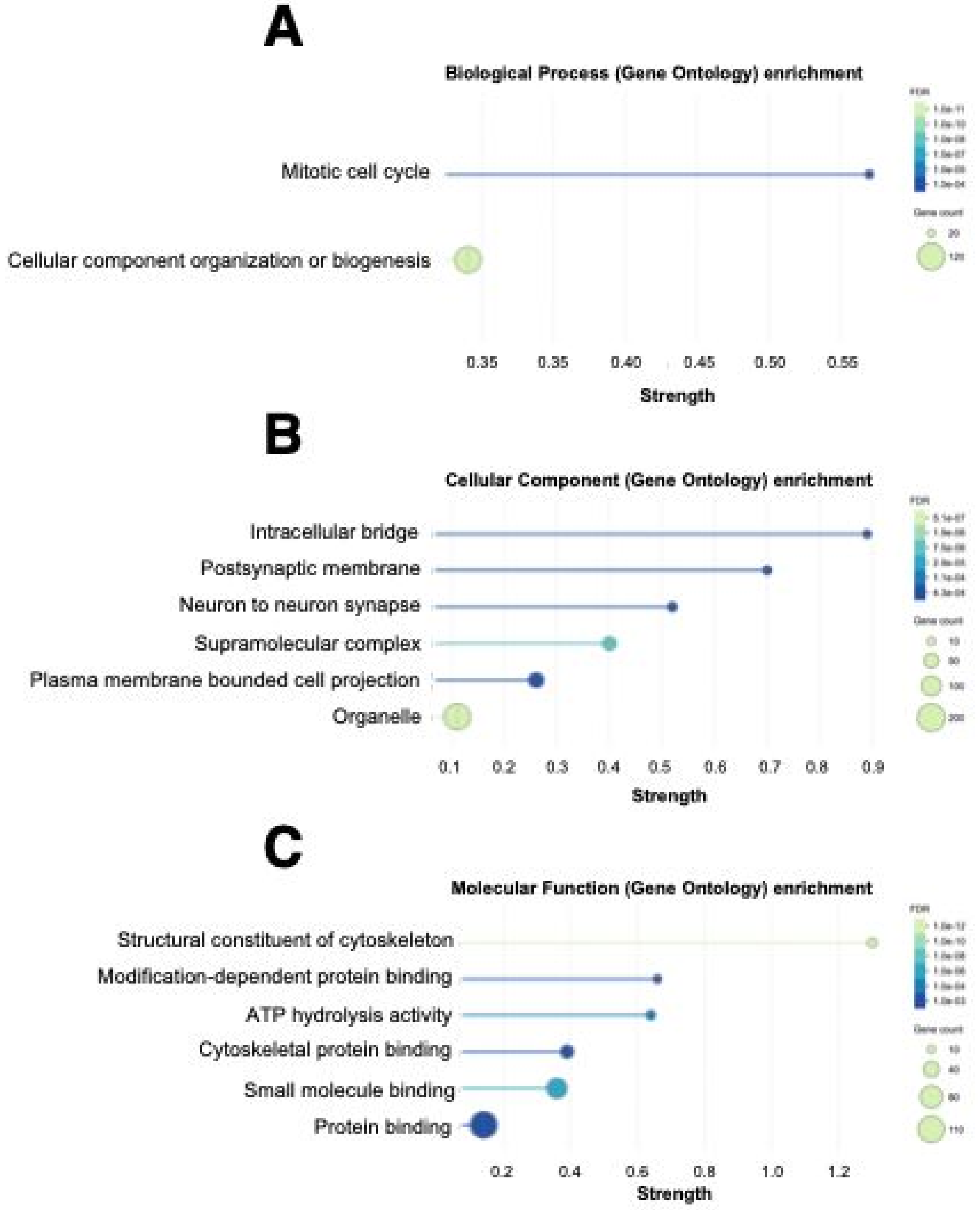

Figure S3. GO enrichment analysis of differentially expressed proteins in nicotine males relative to nicotine females. (A) biological process, (B) cellular component, and (C) molecular function. Bubble size reflects gene count per category; color indicates false discovery rate (FDR). GO enrichment performed using STRING's built-in tool with 0.9 confidence interaction score, FDR < 0.01 and a merge value ≤ 3 to combine related terms.

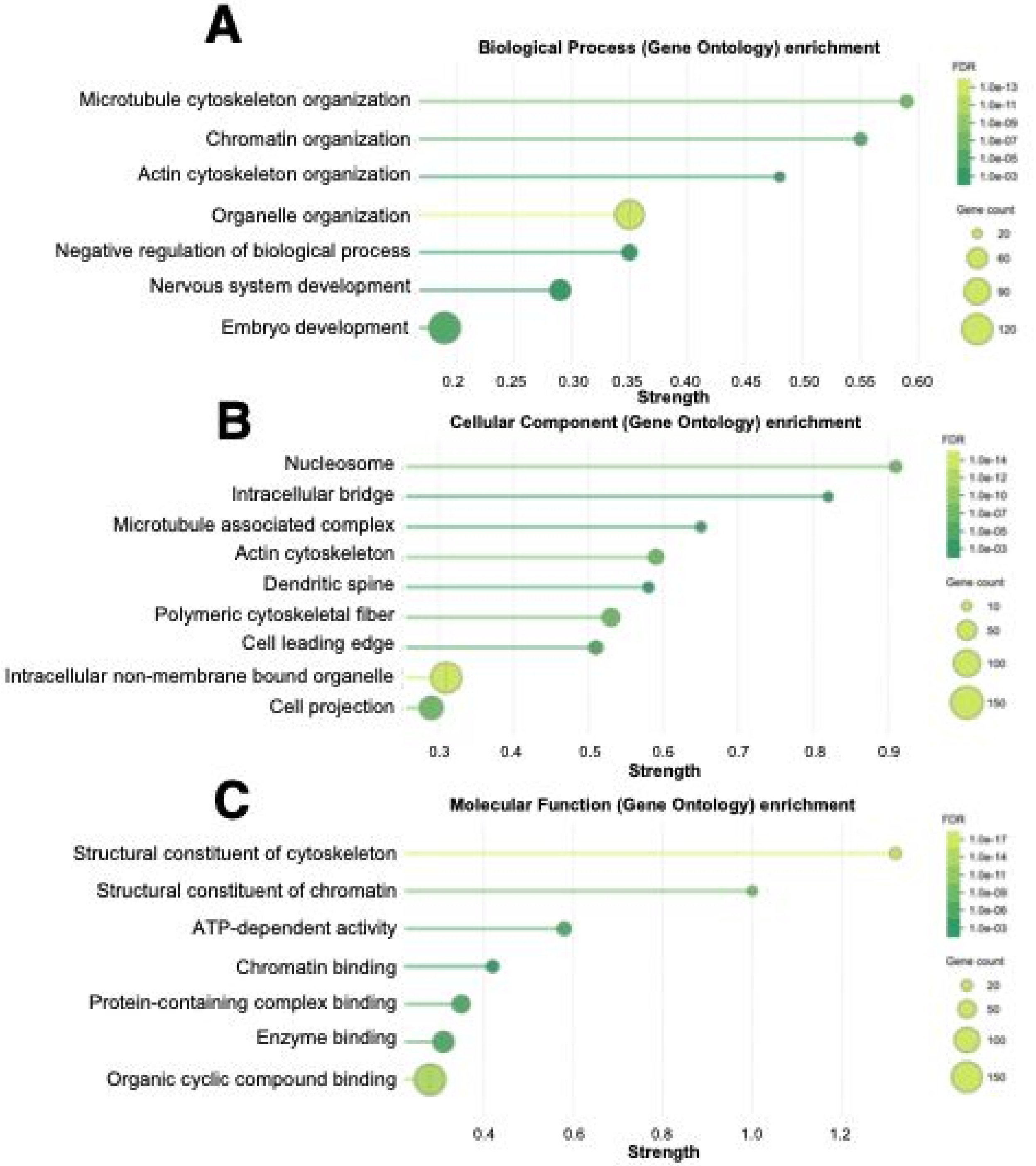

Figure S4. Overlap between sex-dependent microglial proteome differences in control and nicotine-exposed groups. Venn diagram illustrating overlap between proteins significantly altered in control conditions (MC *vs.* FC, n = 374) and proteins significantly differing between sexes following nicotine exposure (MN *vs.* FN). The overlap of 43 proteins represents approximately 14% of the MN *vs.* FN differences, suggesting that the majority of sex-dependent proteomic differences by nicotine are not attributable to baseline sex differences in the microglial proteome.

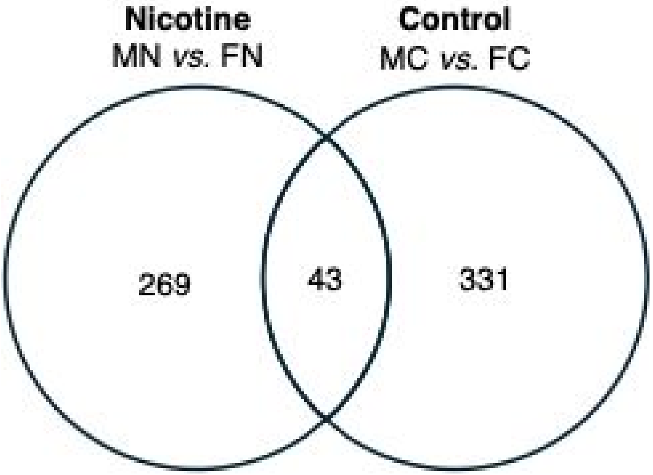

Table S1. Summary of microglia isolation metrics by sex and treatment condition.

	Female control	Female nicotine	Male control	Male nicotine
**Pooled tissue weight (mg)**	500	900	600	800
**Microglia ($CD11b^{high}$ $CD45^{low/intermediate}$)**	40,000	97,000	79,000	72,000
**% Microglia**	0.37%	0.31%	0.52%	0.41%
**% Circulating monocytes ($CD11b^{high}$ $CD45^{high}$)**	0.000271%	0.00062%	0.00098%	0.0018%
**Microglia CD11b MFI**	53,271	54,545	53,683	54,135
**Microglia protein concentration (µg/ul)**	1.338	1.453	1.424	1.500
